# Effectiveness of potential antiviral treatments in COVID-19 transmission control: a modelling study

**DOI:** 10.1186/s40249-021-00835-2

**Published:** 2021-04-19

**Authors:** Sheng-Nan Lin, Jia Rui, Qiu-Ping Chen, Bin Zhao, Shan-Shan Yu, Zhuo-Yang Li, Ze-Yu Zhao, Yao Wang, Yuan-Zhao Zhu, Jing-Wen Xu, Meng Yang, Xing-Chun Liu, Tian-Long Yang, Li Luo, Bin Deng, Jie-Feng Huang, Chan Liu, Pei-Hua Li, Wei-Kang Liu, Fang Xie, Yong Chen, Yan-Hua Su, Ben-Hua Zhao, Yi-Chen Chiang, Tian-Mu Chen

**Affiliations:** 1 State Key Laboratory of Molecular Vaccinology and Molecular Diagnostics, School of Public Health, Xiamen University, 4221-117 South Xiang’an Road, Xiang’an, Xiamen, Fujian People’s Republic of China; 2 Medical Insurance Office, Xiang’an Hospital of Xiamen University, Xiamen, Fujian People’s Republic of China; 3 Clinical Medical Laboratory, Xiang’an Hospital of Xiamen University, Xiamen, Fujian People’s Republic of China; 4 Department of Stomatology, School of Medicine, Xiamen University, Xiamen, Fujian People’s Republic of China

**Keywords:** COVID-19, Antiviral treatment, Age group, Transmission model

## Abstract

**Background:**

Novel coronavirus disease 2019 (COVID-19) causes an immense disease burden. Although public health countermeasures effectively controlled the epidemic in China, non-pharmaceutical interventions can neither be maintained indefinitely nor conveniently implemented globally. Vaccination is mainly used to prevent COVID-19, and most current antiviral treatment evaluations focus on clinical efficacy. Therefore, we conducted population-based simulations to assess antiviral treatment effectiveness among different age groups based on its clinical efficacy.

**Methods:**

We collected COVID-19 data of Wuhan City from published literature and established a database (from 2 December 2019 to 16 March 2020). We developed an age-specific model to evaluate the effectiveness of antiviral treatment in patients with COVID-19. Efficacy was divided into three types: (1) viral activity reduction, reflected as transmission rate decrease [reduction was set as *v* (0–0.8) to simulate hypothetical antiviral treatments]; (2) reduction in the duration time from symptom onset to patient recovery/removal, reflected as a 1/*γ* decrease (reduction was set as 1–3 days to simulate hypothetical or real-life antiviral treatments, and the time of asymptomatic was reduced by the same proportion); (3) fatality rate reduction in severely ill patients (*f*_*c*_) [reduction (*z*) was set as 0.3 to simulate real-life antiviral treatments]. The population was divided into four age groups (groups 1, 2, 3 and 4), which included those aged ≤ 14; 15–44; 45–64; and ≥ 65 years, respectively. Evaluation indices were based on outbreak duration, cumulative number of cases, total attack rate (TAR), peak date, number of peak cases, and case fatality rate (*f*).

**Results:**

Comparing the simulation results of combination and single medication therapy s, all four age groups showed better results with combination medication. When 1/*γ* = 2 and v = 0.4, age group 2 had the highest TAR reduction rate (98.48%, 56.01–0.85%). When 1/*γ* = 2, *z* = 0.3, and v = 0.1, age group 1 had the highest reduction rate of *f* (83.08%, 0.71–0.12%).

**Conclusions:**

Antiviral treatments are more effective in COVID-19 transmission control than in mortality reduction. Overall, antiviral treatments were more effective in younger age groups, while older age groups showed higher COVID-19 prevalence and mortality. Therefore, physicians should pay more attention to prevention of viral spread and patients deaths when providing antiviral treatments to patients of older age groups.
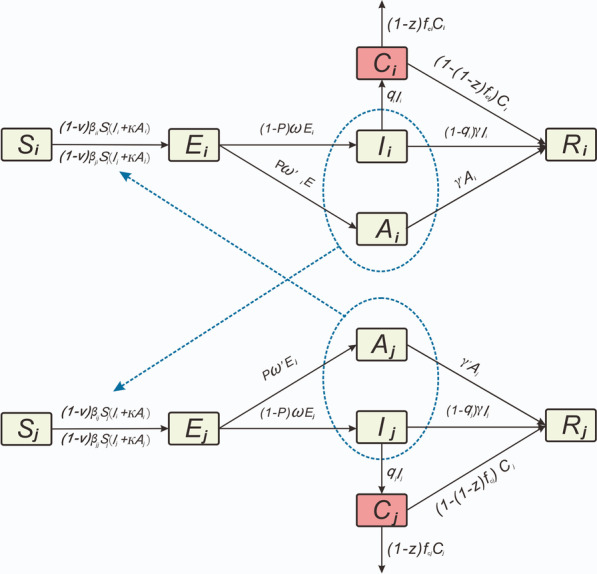

**Supplementary Information:**

The online version contains supplementary material available at 10.1186/s40249-021-00835-2.

## Background

On January 30, 2020, the World Health Organization (WHO) declared novel coronavirus disease 2019 (COVID-19) a public health emergency of international concern [1] and a challenging public health crisis [2]. Many studies have reported that COVID-19 has caused an enormous disease burden globally [3, 4], and it continues to spread vigorously in the United States, Brazil, and other countries [5]. Presently, international epidemic prevention and control strategies for COVID-19 include pharmaceutical interventions (PIs) (antiviral treatment and vaccination) and non-pharmaceutical interventions (NPIs) [isolation, wearing of masks, social distancing (closing of schools, cancellation of collective activities, and crowd gathering restrictions), and tourism restrictions]. The effectiveness of NPIs have been proven in many countries [6–13]. China, especially, successfully controlled the first wave of the COVID-19 outbreak by strictly implementing various public health policies, including the timely isolation of cases, contact tracing, social distancing control, and entertainment activity restriction [12, 14–16]. Furthermore, Republic of Korea's timely public health measures also achieved remarkable results in epidemic control [17].

NPIs implemented were dependent on a variety of factors, such as social and economic systems and required good cooperation from the public. Further, ‘lockdowns’ produced substantial economic hardship [18]. Meanwhile, model analysis showed that characteristics of severe acute respiratory syndrome coronavirus 2 (SARS-CoV-2) allow it to stably coexist with people [19]. When SARS-CoV-2 reaches the endemic phase, its overall pathogenicity in the population may be equivalent to that of common cold viruses [19]. NPIs can neither be maintained indefinitely nor conveniently implemented in all countries. Furthermore, evidence has already indicated that these types of public health measures alone may be insufficient for controlling COVID-19 [20].

As work gradually resumes, and productivity increases, various provinces and cities in China are facing the potential risk of an additional wave of COVID-19 outbreak [21]. Since the virus continues to spread, PIs will be essential for preventing and controlling the spread of COVID-19 [14, 22], thereby reducing social control dependence [23]. Recently, numerous studies have been published on the developmental process, safety, and efficacy of COVID-19 vaccines [24–30]. Moreover, our team has conducted relevant research on this subject [31]. However, the primary role of vaccination is to prevent COVID-19.

Approximately 2000 ongoing trials on the efficacy of pharmacological therapy against SARS-CoV-2 infection have been registered in the WHO International Clinical Trials Registry Platform. Nevertheless, no specific drug has been confirmed to be effective [32]. A systematic review and network meta-analysis of the efficacy and safety of 31 existing drugs revealed that anti-inflammatory agents (tocilizumab, anakinra, and gamma globulin) and remdesivir might improve the prognosis of patients with COVID-19. Furthermore, hydroxychloroquine may be associated with cardiac and non-cardiac safety risks after clearing the virus [33], and dexamethasone reduces the 28-day mortality risk in severely ill patients, especially in those receiving invasive ventilation [34]. The efficacy of antiviral treatments requires further verification, and most recent studies evaluating antiviral treatments focus on clinical efficacy specifically. Further, many studies have shown that the prevalence and case fatality rate of COVID-19 varies among different age groups [15, 35–38]. Therefore, we aimed to evaluate the effectiveness of COVID-19 antiviral treatments among different age groups based on the hypothetical or real-life efficacy of antiviral treatments from the public health perspective. On the one hand, we hope to predict the public health effects of existing antiviral treatments; on the other hand, we hope to provide a model that can be directly applied to evaluate the effectiveness of novel antiviral treatments based on their clinical efficacy, when such antiviral treatments becomes available in the future.

## Methods

### Data source and study design

The following previously published data were collected from patients with COVID-19 in Wuhan from 2 December 2019 to 16 March 2020: age, clinical severity classification (mild, moderate, severe, and critical), date of onset, and date of death [31]. The study population was divided into four age groups based on previous study findings [31, 36, 39], as follows: group 1, ≤ 14 years; group 2, 15–44 years; group 3, 45–64 years; and group 4, ≥ 65 years. The total population in Wuhan City at the time of the prior study was 11 080 996 for those aged ≤ 14, 15–44, 45–64, and ≥ 65 years, the number of individuals in Wuhan city was 1 256 552; 5 210 885; 3 374 388; and 1 239 171, respectively [31].

### Age-specific model

An age-specific Susceptible–exposed–symptomatic–asymptomatic-recovered/removed (SEIAR) model was developed previously [31, 39]. To implement the model, individuals were divided into the following five categories: susceptible (*S*), exposed (*E*), symptomatic (*I*), asymptomatic (*A*), and recovered/removed (*R*). The rate of infection transmissibility for each age group was estimated using the model, and the process of data fitting detailed previously [31] depended on the following assumptions:

1. *S* individuals in age group *i* (the range of *i* and *j* was 1–4, indicating different age groups) were infected by exposure to *I* and *A* individuals in age group *i* and other age groups. The transmission rates of *S* were *β* and *κβ* (0 < *κ* < 1) after effective contacts with *I* and *A*, respectively.
2. The transmission rate (*β*) was divided into two categories: *β*_*ii*_ (within the age group *i*) and *β*_*ij*_ (age groups *i* to *j*).
3. Parameter *p* was set as the proportion of *A* individuals, whereas incubation and latent periods of *I* and *A *were 1/*ω* and 1/*ω′*, respectively.
4. The times from categories *I* and *A* to category *R* were set as 1/*γ* and 1/*γ′*, respectively.
5. The case fatality rate was set as *f* for members of category *I* who died after infection.

The flowchart of this model is presented in our previously published paper, as well as some parameter estimations, such as *k*, *p*, *ω*, *ω′*, *γ*, and *γ′* [31]. The equations used in the current model are as follows:

$$i\ne j$$

$$\frac{d{S}_{i}}{dt}=-{\beta }_{ii}{S}_{i}\left({I}_{i}+\kappa {A}_{i}\right)-{\beta }_{ji}{S}_{i}\left({I}_{j}+\kappa {A}_{j}\right)$$

$$\frac{d{E}_{i}}{dt}={\beta }_{ii}{S}_{i}\left({I}_{i}+\kappa {A}_{i}\right)+{\beta }_{ji}{S}_{i}\left({I}_{j}+\kappa {A}_{j}\right)-\left(1-p\right)\omega {E}_{i}-p{\omega }^{^{\prime}}{E}_{i}$$

$$\frac{d{I}_{i}}{dt}=\left(1-p\right)\omega {E}_{i}-\gamma {I}_{i}-{f}_{i}{I}_{i}$$

$$\frac{d{A}_{i}}{dt}=p{\omega }^{^{\prime}}{E}_{i}-{\gamma }^{^{\prime}}{A}_{i}$$

$$\frac{d{R}_{i}}{dt}={\gamma I}_{i}+{{\gamma }^{^{\prime}}A}_{i}$$

$$\frac{d{S}_{j}}{dt}=-{\beta }_{jj}{S}_{j}\left({I}_{j}+\kappa {A}_{j}\right)-{\beta }_{ji}{S}_{j}\left({I}_{i}+\kappa {A}_{i}\right)$$

$$\frac{d{E}_{j}}{dt}={\beta }_{jj}{S}_{j}\left({I}_{j}+\kappa {A}_{j}\right)+{\beta }_{ji}{S}_{j}\left({I}_{i}+\kappa {A}_{i}\right)-\left(1-p\right)\omega {E}_{j}-p{\omega }^{^{\prime}}{E}_{j}$$

$$\frac{d{I}_{j}}{dt}=\left(1-p\right)\omega {E}_{j}-\gamma {I}_{j}-{f}_{j}{I}_{j}$$

$$\frac{d{A}_{j}}{dt}=p{\omega }^{^{\prime}}{E}_{j}-{\gamma }^{^{\prime}}{A}_{j}$$

$$\frac{d{R}_{j}}{dt}={\gamma I}_{j}+{{\gamma }^{^{\prime}}A}_{j}$$

$$N={S}_{i}+{E}_{i}+{I}_{i}+{A}_{i}+{R}_{i}$$

With subscripts *i* and *j* (*i* ≠ *j*) representing age groups 1–4.

Based on the existing data stage grouping, we performed data fitting and calculated the dissemination capacity of the four age groups (stages 1, 2, 3, and 4 occurred from 2 December 2019 to 23 January 2020; 24 January to 2 February 2020; 3 February to 18 February 2020; and 19 February 2020 to 16 March 2020, respectively; the details of which are shown in our previous article [31]). The results of data fitting are shown in Additional file 1: Fig. S1 and Additional file 3: Table S1.

### Age-specific model for antiviral treatments

**Fig. 1:**
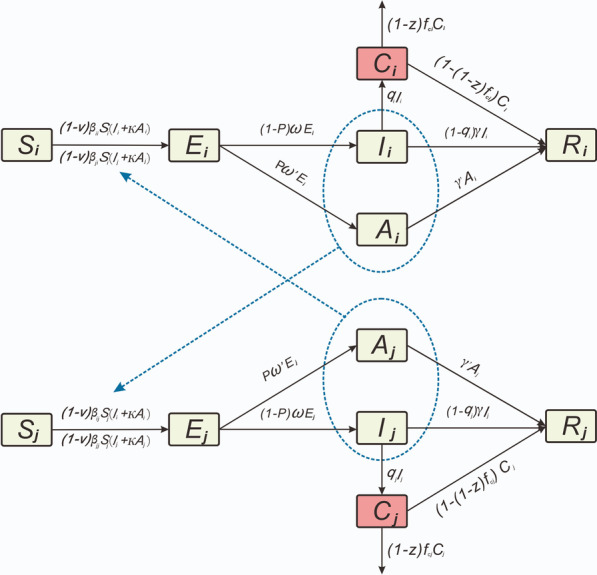
The framework of Susceptible–Exposed–Symptomatic–Asymptomatic-Recovered/Removed (SEIAR) model

Firstly, death only occurred in severely ill patients [15], therefore, we classified all patients as either severely (severe and critical) or non-severely ill (mild and moderate), according to the COVID-19 clinical severity classification of each patient. Based on the existing age-specific model [39, 40], we distinguished severely ill patients from category *I* patients. The framework of the SEIAR model for antiviral treatment is shown in Fig. 1. In this model, the population was divided into six categories, as follows: susceptible (*S*), exposed (*E*), symptomatic (*I*), severely ill patients (*C*), asymptomatic (*A*), and recovered/removed (*R*). When implementing the model, the following assumptions were made:

1. *S* individuals in age group *i* (the range of *i* and *j* was 1–4, indicating different age groups) were infected by exposure to *I* and *A* individuals in age group *i* and other age groups. The transmission rates of *S* were *β* and *κβ* (0 < *κ* < 1) after effective contacts with *I* and *A*, respectively.
2. The transmission rate (*β*) was divided into two categories, as follows: *β*_*ii*_ (within the age group *i*) and *β*_*ij*_ (age group *i* to *j*), and the reduction ratio of *β* was set as *v* (the initial value of *v* was 0).
3. Parameter *p* was set as the proportion of *A* individuals, whereas the incubation and latent periods of *I* and *A* were 1/*ω* and 1/*ω′*, respectively.
4. The ratio of severely ill patients in age group *i* was set as *q*_*i*_, and the fatality rate of severely ill patients in age group *i* was set as *f*_*ci*_. The reduction ratio of *f*_*ci*_ was set as *z* (initial value of *z* was 0), and the number of people who changed from *C*_*i*_ to *R*_*i*_ was (1 − [1 − *z*] *f*_*ci*_) *C*_*i*_ at time *t*.
5. Durations needed to change from categories *I* and *A* to category *R* were set as 1/*γ* and 1/*γ′*, respectively. Therefore, the numbers of people who transitioned from *I* to *R* and *A* to *R*, respectively, were *γI* and *γ′A* at time *t*.

The following equations were used in the model:

$$i\ne j$$

$$\frac{d{S}_{i}}{dt}=-(1-\mathrm{v}){\beta }_{ii}{S}_{i}\left({I}_{i}+\kappa {A}_{i}\right)-(1-\mathrm{v}){\beta }_{ji}{S}_{i}\left({I}_{j}+\kappa {A}_{j}\right)$$

$$\frac{{dE_{i} }}{dt} = \left( {1 - v} \right)\beta_{ii} S_{i} \left( {I_{i} + \kappa A_{i} } \right) + \left( {1 - v} \right)\beta_{ji} S_{i} \left( {I_{j} + \kappa A_{j} } \right) - \left( {1 - p} \right)\omega E_{i} - p\omega^{\prime}E_{i}$$

$$\frac{{dA_{i} }}{dt} = p\omega^{\prime}E_{i} - \gamma^{\prime}A_{i}$$

$$\frac{d{I}_{i}}{dt}=(1-p)\omega {E}_{i}-{q}_{i}{I}_{i}-(1-{q}_{i})\gamma {I}_{i}$$

$$\frac{d{C}_{i}}{dt}={q}_{i}{I}_{i}-(1-z){f}_{ci}{C}_{i}-(1-\left(1-\mathrm{z}\right){f}_{ci}){C}_{i}$$

$$\frac{d{R}_{i}}{dt}=\left(1-\left(1-\mathrm{z}\right){f}_{ci}\right){C}_{i}+\left(1-{q}_{i}\right)\gamma {I}_{i}+{\gamma }^{^{\prime}}{A}_{i}$$

$${X}_{i}=(1-p)\omega {E}_{i}$$

$$\frac{d{S}_{j}}{dt}=-(1-\mathrm{v}){\beta }_{jj}{S}_{j}\left({I}_{j}+\kappa {A}_{j}\right)-(1-\mathrm{v}){\beta }_{ij}{S}_{j}\left({I}_{i}+\kappa {A}_{i}\right)$$

$$\frac{{dE_{j} }}{dt} = \left( {1 - v} \right)\beta_{jj} S_{j} \left( {I_{j} + \kappa A_{j} } \right) + \left( {1 - v} \right)\beta_{ij} S_{j} \left( {I_{i} + \kappa A_{i} } \right) - \left( {1 - p} \right)\omega E_{j} - p\omega^{\prime}E_{j}$$

$$\frac{{dA_{j} }}{dt} = p\omega^{\prime}E_{j} - \gamma^{\prime}A_{j}$$

$$\frac{d{I}_{j}}{dt}=(1-p)\omega {E}_{j}-{q}_{j}{I}_{j}-(1-{q}_{j})\gamma {I}_{j}$$

$$\frac{d{C}_{j}}{dt}={q}_{j}{I}_{j}-(1-z){f}_{cj}{C}_{j}-(1-\left(1-\mathrm{z}\right){f}_{cj}){C}_{j}$$

$$\frac{d{R}_{j}}{dt}=\left(1-\left(1-\mathrm{z}\right){f}_{cj}\right){C}_{j}+\left(1-{q}_{j}\right)\gamma {I}_{j}+{\gamma }^{^{\prime}}{A}_{j}$$

$${X}_{j}=(1-p)\omega {E}_{j}$$

$$N={S}_{i}+{E}_{i}+{I}_{i}+{A}_{i}+{R}_{i}+{S}_{j}+{E}_{j}+{I}_{j}+{A}_{j}+{R}_{j}$$

### Parameter estimation

Parameter values used in the model and the methods used for their determination are detailed in Table 1. The model had 30 parameters (*β*_*ii*_, *β*_*ij*_, *β*_*jj*_, *β*_*ji*_, *f*_*ci*_, *q*_*i*_, *κ*, *p*, *ω*, *ω′*, *γ* and *γ′*).

**Fig. 2:**
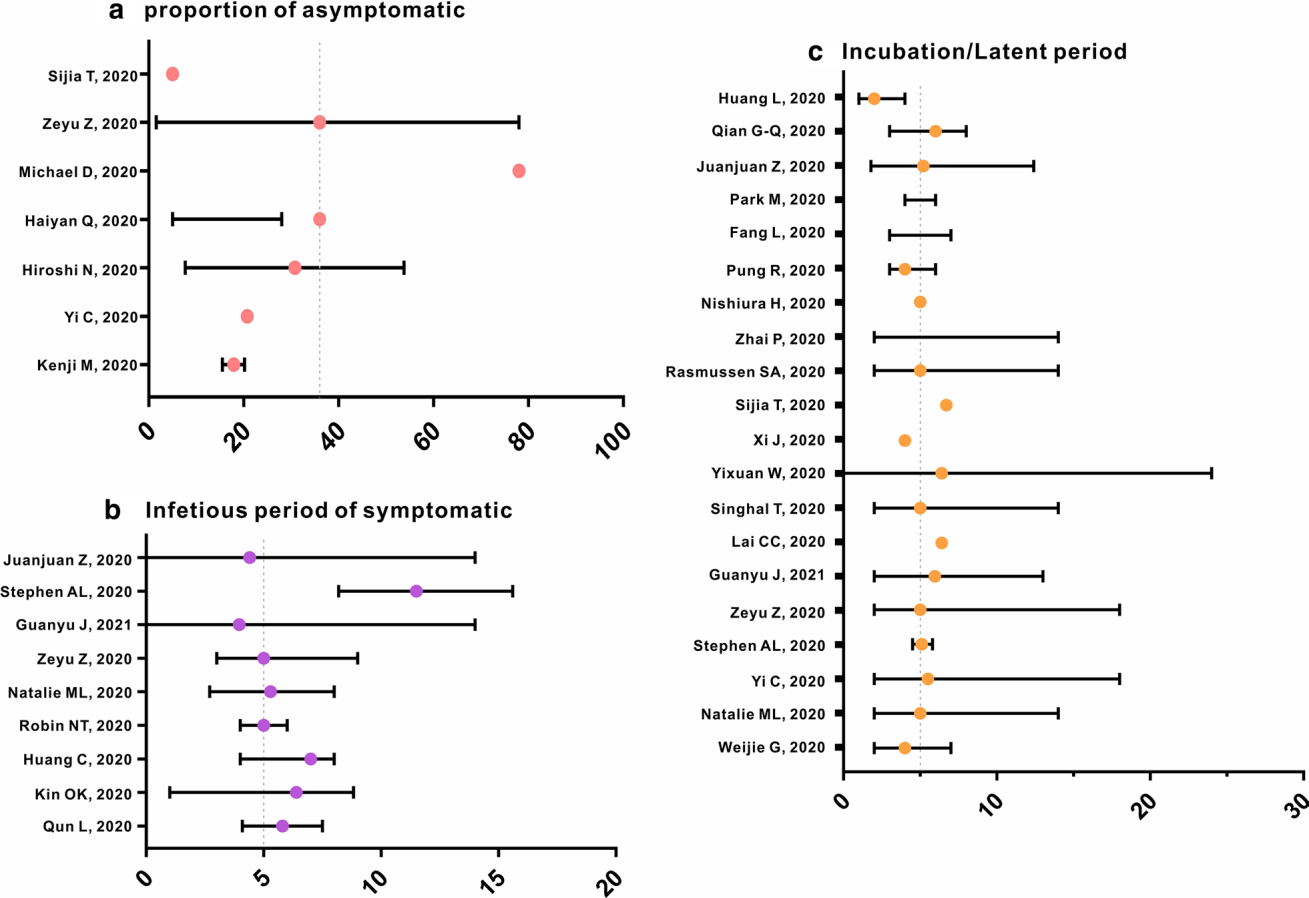
Summary of reported parameters of *p*, *ω*, *γ.* **a** Reference about proportion of asymptomatic. **b** Reference about infectious period of asymptomatic. **c** Reference about incubation/latent period

1. We used an age-specific model to fit data, and obtained results of four stages. The parameters of *β* that we used for the simulation were the results determined during stage 1 (Additional file 2: Fig. S2).
2. Based on the analysis of data, ratios of severely ill patients (*q*_*i*_) were 3.25%, 10.99%, 19.14%, and 37.79% for age groups 1, 2, 3, and 4, respectively. Fatality rates for severely ill patients (*f*_*c*_) of the groups were 5.00%, 5.16%, 18.20%, and 39.79%, respectively.
3. A previous study indicated that 4.11% and 6.30% of individuals would become infected after close contact with *A* and *I* patients, respectively [41]. Moreover, in the study, *k* was set as 0.65 [31]; therefore, in this study, we assumed that the transmissibility of *A* infections was 0.65 times that of *I* infections. Further, it has been reported that the transmissibility of *I* is 3.9 times that of *A*, and an asymptomatic individual may infect 11 people [42]. Another study set *κ* as 1.0 [39]. Based on previous results, we set the range of *k* to 0–1.
4. Values of *p* used in different studies have varied. For instance, the proportion of *A* patients in the Diamond Princess cruise ship was 17.9% [95% confidence interval (*CI*): 15.5–20.2%] [43] and 20.75% in Ningbo City [41]. In addition, a study estimated an *A* ratio of 30.8% (95% *CI*: 7.7–53.8%) using a binomial distribution [44], whereas another study reported that *A* patients constituted 5% to 28% of all patients with COVID-19 [35]. Furthermore, it was also reported that the *A* ratio could reach 78% [45]. Therefore, since we set the *A* proportion (*p*) to 0.36 previously (1.6–78%) [31], we used the same value in the present study (Fig. 2-a).
5. A previous study revealed that the incubation period early in the epidemics in Wuhan City was 4 days (interquartile range: 2–7 days) [46], whereas other studies indicated that the incubation period in Wuhan [47] and Ningbo [41] raged from 2–14 days and 2–18 days, respectively. In addition, another study reported an incubation period of 5.1 days (95% *CI*: 4.5–5.8) [48]. Therefore, we set the incubation period to 5 days previously (*ω* = *ω*’ = 0.2) based on the median incubation periods of multiple studies [31]. The same value was used in the present study. We show incubation periods reported in many different studies in Fig. 2c, and, based on current findings, determined that setting the incubation period as 5 days is representative of published data.
6. Various studies have reported the following durations from symptom onset to hospitalisation: 6.39 (range: 1.00–8.83), 7, 4–6, and 4.1–7.5 days [36, 49–51]. Moreover, right-truncated data indicated that the time from illness onset to hospitalisation ranged from 2.7 to 8.0 days [47]. Therefore, in our previous study, the infectious periods for *I* patients were set to 5 days (*γ* = 0.2) [31]. The same value, which was based on the median infectious period determined in multiple studies, was used in the present study. We show infectious periods of *I* determined previously in Fig. 2-b. A prior study indicated that the median infectious period of 24 *A* patients was 9.5 days (range: 1–21 days) [52]. Therefore, we set *γ’* as 0.1 in the model [31].

**Table 1:** Description and values of parameters in the age-specific SEICAR model

Parameter	Description	Unit	Value	Range	Method
*β*_ii_^*^	Transmission relative rate among age group *i*	Individuals^−1^·days^−1^	Shown in text	≥ 0	Curve fitting
*β*_ij_^*^	Transmission relative rate from age group *i* to *j*	Individuals^−1^·days^−1^	Shown in text	≥ 0	Curve fitting
*β*_ji_^*^	Transmission relative rate from age group *j* to *i*	Individuals^−1^·days^−1^	Shown in text	≥ 0	Curve fitting
*β*_jj_^*^	Transmission relative rate among age group *i*	Individuals^−1^·days^−1^	Shown in text	≥ 0	Curve fitting
*κ*	Relative transmissibility rate of asymptomatic to symptomatic individuals	1	0.65	0–1	Refs. [31, 39, 41, 42]
*p*	Proportion of the asymptomatic	1	0.36	0.016–0.78	Refs. [31, 35, 41, 43–45]
*ω*	Incubation relative rate	days^−1^	0.2	0.05556–0.5	Refs. [31, 41, 46–48]
*ω*′	Latent relative rate	days^−1^	0.2	0.05556–0.5	Refs. [31, 41, 46–48]
*q*_i_^*^/*q*_j_^*^	Severely rate of symptomatic in age group *i/j*	1	Shown in text	≥ 0	Analysis of data
*γ*	Recovered/Removed rate of the symptomatic	days^−1^	0.2	0.1111–0.3333	Refs. [31, 36, 47, 49–51]
*γ′*	Recovered/Removed rate of the asymptomatic	days^−1^	0.1	0.04762–1	Refs. [31, 52]
*f*_ci_^*^/ *f*_cj_^*^	Fatality of the disease in age group *i/j*	1	Shown in text	≥ 0	Analysis of data

^*^i and *j* represent age group 1 to 4, respectively, and *i* ≠ *j*

### Antiviral treatment simulation

Existing antiviral treatments and their clinical efficacies are shown in Table 2. Based on current research, we divided the effectiveness of antiviral treatment into three types: (1) reducing transmission (*β*); (2) decreasing the infectiousness of *I* and *A* (1/*γ* and 1/*γ’*); and (3) reducing the fatality rate of severely ill patients (*f*_*c*_).

1. A prior study showed that remdesivir efficiently inhibited viral infection in a human cell line (human liver cancer Huh-7 cells) sensitive to SARS-CoV-2; thus, remdesivir can reduce the infectivity of COVID-19 to an extent [46]. Similarly, researchers determined that chloroquine has antiviral activity and can synergistically enhance antiviral effects of remdesivir in vivo [46]. In the model, antiviral treatments capable of reducing viral activity were revealed by decreases in *β*. However, existing studies have not specified the extent to which antiviral treatments reduce *β* values, and therefore, the reduction of *β* was determined by adjusting the value of *v* (0*–*0.8) to simulate that of hypothetical antiviral treatments.
2. Previous studies have shown that remdesivir might not improve recovery or reduce mortality in patients with COVID-19 [53, 54]. However, a preliminary study in the United States, involving many hospitals globally, demonstrated that using remdesivir in hospitalised patients with COVID-19 receiving oxygen therapy shortened their recovery time from 15 to 11 days and reduced risk of respiratory infections [55]. Some clinical trials have shown that the recovery time of patients with COVID-19 who use remdesivir is 31% faster than that of patients provided a placebo [56]. For severely ill patients, combined use of ribavirin with interferon beta-1b and lopinavir/ritonavir safely and efficiently shortens the duration and reduce symptoms of viral infection in patients with mild-to-moderate disease, with mild side effects [57]. In addition, other studies have shown that chloroquine phosphate can shorten the clinical course of COVID-19 [36]. In this model, antiviral treatments capable of reducing the duration needed to transition from categories *I* and *A* to category *R* were reflected in 1/*γ* and 1/*γ′* decrease. The reduced duration needed to transition from category *I* was set to 1–3 days to simulate hypothetical or real-life antiviral treatments, and the duration from category *A* was reduced proportionally to 1/*γ*.
3. Dexamethasone use reduced the number of deaths among patients with COVID-19 using ventilators by 30% [34, 58]. In addition, the improvement effect of tocilizumab on severely or critical ill patients with COVID-19 has also been confirmed. Futher, interferon-alpha 2b was reported to affect survival in patients with COVID-19 [59–61]. Another research team successfully identified multiple highly active neutralising antibodies in the plasma of recovered patients [62]. Experimental treatments using plasma of recovered patients have also been shown to significantly effective in severely and critically ill patients [63], but their widespread use is not feasible at present. In the model, antiviral treatments capable of reducing fatality rates of severely ill patients were reflected as decreases in *f*_*c*_ (reduction was set as *z*). We set *z* as 0.3, to simulate real-life antiviral treatment.

**Table 2:** Summary of existing antiviral treatments

No.	Author	Year	Title	Antiviral treatment	Clinical efficacy	Statistical significance	Effectiveness
1	Wang M-L, Cao R-Y, Zhang L-K, et al.	2020	Remdesivir and chloroquine effectively inhibit the recently emerged novel coronavirus (2019-nCoV) in vitro	Remdesivir	Remdesivir can reduce the infectivity of COVID-19 patients	Yes	1) Reducing transmission (*β*)
2	Wang M-L, Cao R-Y, Zhang L-K, et al.	2020	Remdesivir and chloroquine effectively inhibit the recently emerged novel coronavirus (2019-nCoV) in vitro	Chloroquine	Chloroquine has certain antiviral activity and can synergize with Remdesivir in the body to enhance its antiviral effect	Yes	1) Reducing transmission (*β*)
3	Gautret P, Lagier J-C, Honore S, et al.	2020	Hydroxychloroquine and azithromycin as a treatment of COVID-19: results of an open-label non-randomized clinical trial	Hydroxychloroquine	The average survival time of viral vectors in the treatment group was shorter than that in the control group	Yes	2) Decreasing the infectiousness of I and A (1/*γ* and 1/*γ’*);
4	Million M, Lagier J-C, Gautret P, et al.	2020	Early treatment of COVID-19 patients with hydroxychloroquine and azithromycin: a retrospective analysis of 1061 cases in Marseille	Hydroxychloroquine + Azithromycin	The combined medication can shorten the course of the patient’s disease, and 91.7% of the patients successfully cleared the virus within 10 days	Yes	2) Decreasing the infectiousness of I and A (1/*γ* and 1/*γ’*);
5	Chen Z-W, Hu J-J, Zhang Z-W, et al.	2020	Efficacy of hydroxychloroquine in patients with COVID-19: results of a randomized clinical trial	Hydroxychloroquine	The recovery time of body temperature and cough relief in treatment group were significantly shortened	Yes	2) Decreasing the infectiousness of I and A (1/*γ* and 1/*γ’*);
6	Bian H-J, Zheng Z-H, Wei D, et al.	2020	Meplazumab treats COVID-19 pneumonia: an open-labelled, concurrent controlled add-on clinical trial	Meplazumab	The virus clearance time of the treatment group and control group was 3 days (1.5–4.5) and 13 days (6.5–19.5), respectively	Yes	2) Decreasing the infectiousness of I and A (1/*γ* and 1/*γ’*);
7	Cai Q-X, Yang M-H, Liu D-J, et al.	2020	Experimental Treatment with Favipiravir for COVID-19: An Open-Label Control Study	Favipiravir	The median duration of the disease in the treatment group was 4 days (2.5–9) while the control group was 11 days (8–13)	Yes	2) Decreasing the infectiousness of I and A (1/*γ* and 1/*γ’*);
8	Beigel J-H, Tomashek K-M, Dodd L-E	2020	Remdesivir for the treatment of COVID-19—preliminary report	Remdesivir	The recovery time of hospitalized patients who need oxygen therapy has been shortened from 15 to 11 days after using Remdesivir	Yes	2) Decreasing the infectiousness of I and A (1/*γ* and 1/*γ’*);
9	Gao J-J, Tian Z-X, Yang X	2020	Breakthrough: Chloroquine phosphate has shown apparent efficacy in treatment of COVID-19 associated pneumonia in clinical studies	Chloroquine phosphate	The duration of the disease in the treatment group was shortened	Yes	2) Decreasing the infectiousness of I and A (1/*γ* and 1/*γ’*);
10	Deftereos S-G, Giannopoulos G, Vrachatis D-A, et al.	2020	Effect of Colchicine vs Standard Care on Cardiac and Inflammatory Biomarkers and Clinical Outcomes in Patients Hospitalized With Coronavirus Disease 2019	Colchicine	The clinical deterioration time of the treatment group was significantly improved	Yes	2) Decreasing the infectiousness of I and A (1/*γ* and 1/*γ’*);
11	Cai Q-X, Yang M-H, Liu D-J, et al.	2020	Experimental treatment with favipiravir for COVID-19: An open-label control study	Favipiravir	The case fatality rate of the treatment group has decreased	Yes	3) Reducing the fatality rate of severely ill patients (*f*_*c*_)
12	Horby P, Lim W-S, Mafham M, et al.	2020	Dexamethasone in Hospitalized Patients with Covid-19—Preliminary Report	Dexamethasone	The use of dexamethasone has reduced the death number of COVID-19 patients who use breathing machine by one third	Yes	3) Reducing the fatality rate of severely ill patients (*f*_*c*_)
13	Xu X-L, Han M-F, Li T-T, et al.	2020	Effective treatment of severe COVID-19 patients with tocilizumab	Tobiximab, Interferon α-2b	Tobiximab can improve the clinical symptoms of severe or critical COVID-19 patients, and Interferon α-2b can improve the survival rate of patients	Yes	3) Reducing the fatality rate of severely ill patients (*f*_*c*_)
14	Pereda R, Daniel González D, Rivero H, et al.	2020	Therapeutic effectiveness of interferon-alpha 2b against COVID-19: the Cuban experience	Interferon α-2b	The cure rate in the treatment group (95.4%) is higher than the cure rate in the control group (26.1%)	Yes	3) Reducing the fatality rate of severely ill patients (*f*_*c*_)
15	Beigel J-H, Tomashek K-M, Dodd L-E	2020	Remdesivir for the Treatment of COVID-19 — Preliminary Report	Remdesivir	The recovery time of the treatment group was 11 days while the control group was 15 days; and the case fatality rate of the treatment group was lower	Yes	2) Decreasing the infectiousness of I and A (1/*γ* and 1/*γ’*); 3) reducing the fatality rate of severely ill patients (*f*_*c*_)
16	Kalil A-C, Patterson T-F, Mehta A-K, et al.	2021	Baricitinib plus Remdesivir for Hospitalized Adults with Covid-19	Baricitinib, Remdesivir	Combination medication could shorten the recovery time and reduce the 28-day mortality rate	Yes	2) Decreasing the infectiousness of I and A (1/*γ* and 1/*γ’*); 3) Reducing the fatality rate of severely ill patients (*f*_*c*_)
17	Chen C, Zhang Y, Huang J-Y, et al.	2020	Favipiravir versus Arbidol for COVID-19: A Randomized Clinical Trial	Uminovir, Favipiravir	The 7-day clinical recovery rate was 55.9% in the group of umminovir, and 71% in the group of favipiravir	Yes	2) Decreasing the infectiousness of I and A (1/*γ* and 1/*γ’*); 3) Reducing the fatality rate of severely ill patients (*f*_*c*_)
18	Tong S, Su Y, Yu Y, et al.	2020	Ribavirin therapy for severe COVID-19: a retrospective cohort study	Ribavirin	The recovery time of patients in the treatment group was 12.8 ± 4.1 days and that of control group was 14.1 ± 3.5 days. The case fatality rate in the treatment group was 17.1%, and the case fatality rate in the control group was 24.6%	Yes	2) Decreasing the infectiousness of I and A (1/*γ* and 1/*γ’*); 3) Reducing the fatality rate of severely ill patients (*f*_*c*_)
19	Zhang Y-Tai, Louisa T, Goh R-M, et al.	2020	Mixed Chinese herbs and Western medicine for novel coronavirus disease 2019 (COVID-19): a mixed method review	Traditional Chinese medicine combined therapy	Traditional Chinese medicine combined treatment can improve symptoms, but there is no significant difference in admission time	No	
20	Wang Y-M, Zhang D-Y, Du G-H, et al.	2020	Remdesivir in adults with severe COVID-19: a randomised,double-blind, placebo-controlled, multicentre trial	Remdesivir	The recovery period and case fatality rate of the treatment group were different from those of the control group, but they were not statistically significant	No	
21	Chen J, Liu D, Liu L	2020	A pilot study of hydroxychloroquine in treatment of patients with common coronavirus disease-19 (COVID-19)	Hydroxychloroquine	There was no statistical difference between the treatment group and the control group in clearing the virus	No	
22	Tang W, Cao Z, Han M	2020	Hydroxychloroquine in patients mainly with mild to moderate COVID-19: an open-label, randomized, controlled trial	Hydroxychloroquine	There was no statistical difference between the treatment group and the control group in clearing the virus	No	
23	Boulware D-R, Pullen M-F, Bangdiwala A-S, et al.	2020	A randomized trial of hydroxychloroquine as postexposure prophylaxis for COVID-19	Hydroxychloroquine	Hydroxychloroquine can not reduce virus activity	No	
24	Li Y-P, Xie Z-W, Lin W-Y, et al.	2020	An exploratory randomized, controlled study on the efficacy and safety of lopinavir/ritonavir or arbidol treating adult patients hospitalized with mild/moderate COVID-19	Lopanovi	The case fatality rate and recovery time of the treatment group was lower than that of the control group, but the difference was not statistically significant	No	
25	Hung I-FN, Lung K-C, Tso E-YK, et al.	2020	Triple combination of interferon beta-1b, lopinavir–ritonavir, and ribavirin in the treatment of patients admitted to hospital with COVID-19: an open-label, randomised, phase 2 trial	Ribavirin, Interferon β-1b, Lopinavir	The recovery time after the combination medication was shortened from 12 to 7 days, but the difference was not statistically significant	No	
26	Cao B, Wang Y, Wen D, et al.	2020	A Trial of Lopinavir–Ritonavir in Adults Hospitalized with Severe Covid-19	Lopinavir–Ritonavir	The case fatality rate of the treatment group was lower than that of the control group, but the difference was not statistically significant	No	
27	Li Y-P, Xie Z-W, Lin W-Y, et al.	2020	Efficacy and Safety of Lopinavir/Ritonavir or Arbidol in Adult Patients with Mild/Moderate COVID-19: An Exploratory Randomized Controlled Trial	Lopinavir/Ritonavir or Arbidol	Patients in the treatment group showed no significant improvement after treatment	No	
28	Tobaiqy M, Alhumaid S, Mutair A-A	2020	Efficacy and Safety of Lopinavir/Ritonavir for Treatment of COVID-19: A Systematic Review and Meta-Analysis	Lopinavir–Ritonavir	Patients in the treatment group showed no significant improvement after treatment	No	

### Evaluation index

The evaluation indices were as follows:

1. Outbreak duration (OD, days): the number of days from the infection of the first patient to the recovery or death of the last patient.
2. Cumulative number of cases (CNC): the number of COVID-19 patients during the outbreak.
3. Total attack rate (TAR): the ratio of the cumulative number of cases to the total population.
4. Peak date (PD): the date on which the maximum number of cases was observed.
5. Number of peak cases (NPC): the number of cases recorded on the peak date.
6. Case fatality rate (*f*): the ratio of the number of deaths to the cumulative number of cases identified during the outbreak.

The values of these indices were the simulated number of cases provided different antiviral treatments rather than the actual number.

### Simulation methods and statistical analysis

Berkeley Madonna 8.3.18 (developed by Robert Macey and George Oster, University of California at Berkeley; copyright ©1993–2001 Robert I. Macey & George F. Oster) was employed for curve fitting and simulation. We used the same simulation methods (Runge–Kutta fourth-order method with the tolerance set to 0.001) as described previously [64–69]. Berkeley Madonna was used to adopt the curve fitting of the least root-mean-square deviation. Goodness of fit was judged by the coefficient of determination (*R*^*2*^) value, which was calculated using IBM SPSS Statistics for Windows, version 21.0 (IBM Corp. Armonk, NY, USA) and Microsoft Office Excel 2016 (Microsoft, Redmond, WA, USA).

### Sensitivity analysis

In this study, two parameters were used to analyse the sensitivity of the model: *k* (0–1) and *p* (0.016–0.78), each was split into 1000 values according to its range. The mean and standard deviation (SD) were calculated for sensitivity analysis.

## Results

### Results of the antiviral treatment simulation

This study evaluated the intervention effect of COVID-19 therapies by simulating several antiviral treatments with different efficacies. Overall, we observed age-dependent differences.

Age group-specific results obtained without intervention indicated that an increase in age was associated with increased TAR and *f*, as well as advanced PD. However, with an increase in age, CNC, NPC, and OD initially increased and then decreased. Changes in *β*, *γ* and*γ’* did not vary. When *β*, *γ*, and*γ’* of each age group remained unchanged, changes in *f*_*c*_ did not affect the trend and severity of the epidemic, but reduced *f*. However, by reducing *β* without changing *f*_*c*_, *γ*, and *γ’*, we observed that CNC, NPC, and TAR continued to decrease, while OD was prolonged, and PD continued to be delayed. Although *f* continued to fall, *β* needed to be reduced by 70% in age group 1 and 80% in age groups 2, 3, and 4 to completely control the epidemic. When *β* and *f*_*c*_ remained unchanged, *γ* and *γ’* increased, and CNC, NPC, and TAR continued to decrease; nevertheless, OD was prolonged, and PD continued to be delayed (Additional file 4: Table S2, Additional file 5: Table S3, Additional file 6: Table S4, Additional file 7: Table S5).

When different antiviral treatments were used, the TAR reduction rate was higher than *f* for all age groups, and the reduction of different age groups varied. When comparing the reduction of TAR with *f* when only one type of antiviral treatment was used, we found that changes in *β* and *γ* had the greatest effects on TAR and *f*, respectively. When the value of *v* was 0.7, age group 2 had the highest TAR reduction rate (95.78%). However, when the value of 1/*γ* was 2, age group 1 had the highest *f* reduction rate (64.56%).

**Fig. 3:**
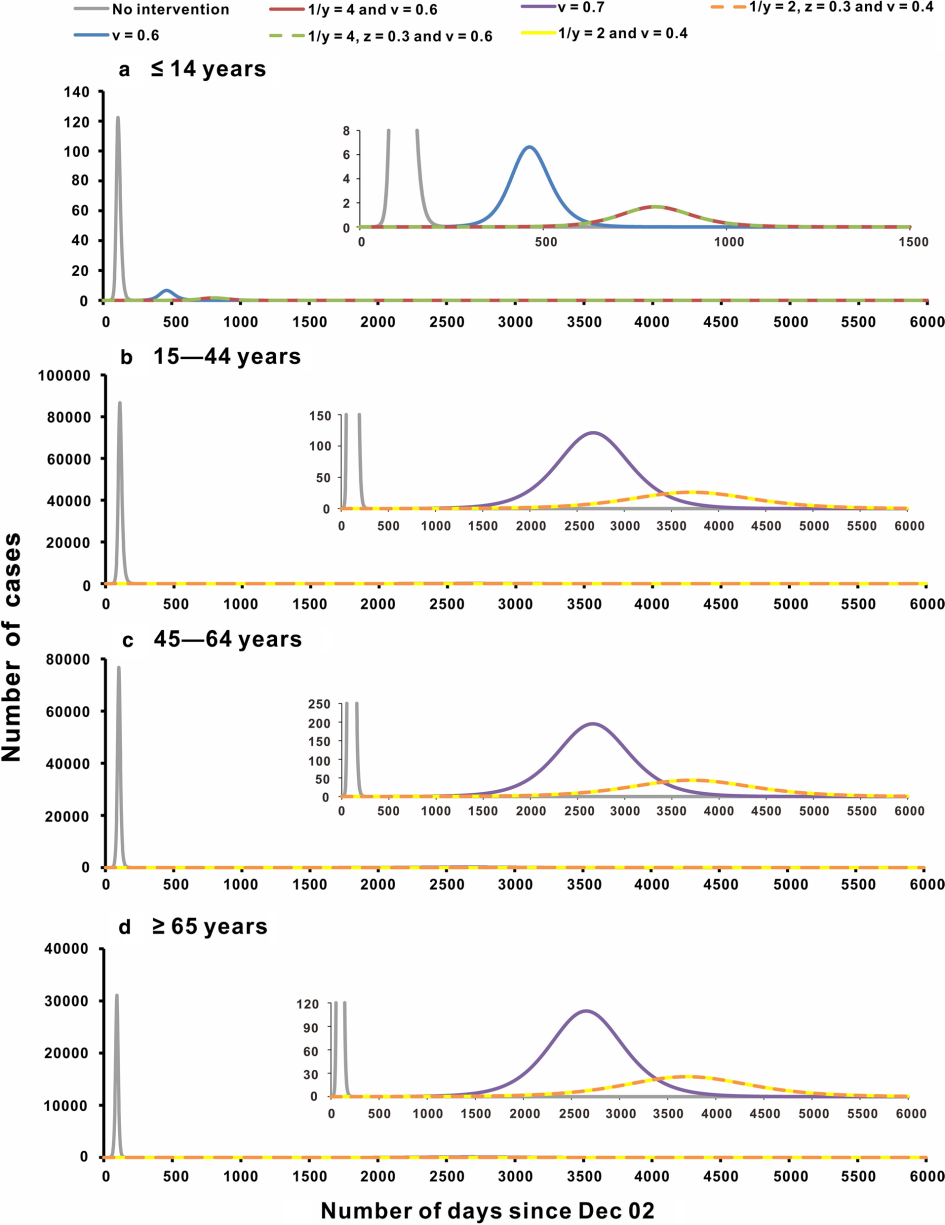
Evaluation of the effectiveness of potential antiviral treatments (with highest TAR reduction in each age group). **a** ≤ 14 years; **b**15–44 years; **c** 45–64 years; **d** ≥ 65 years

**Fig. 4:**
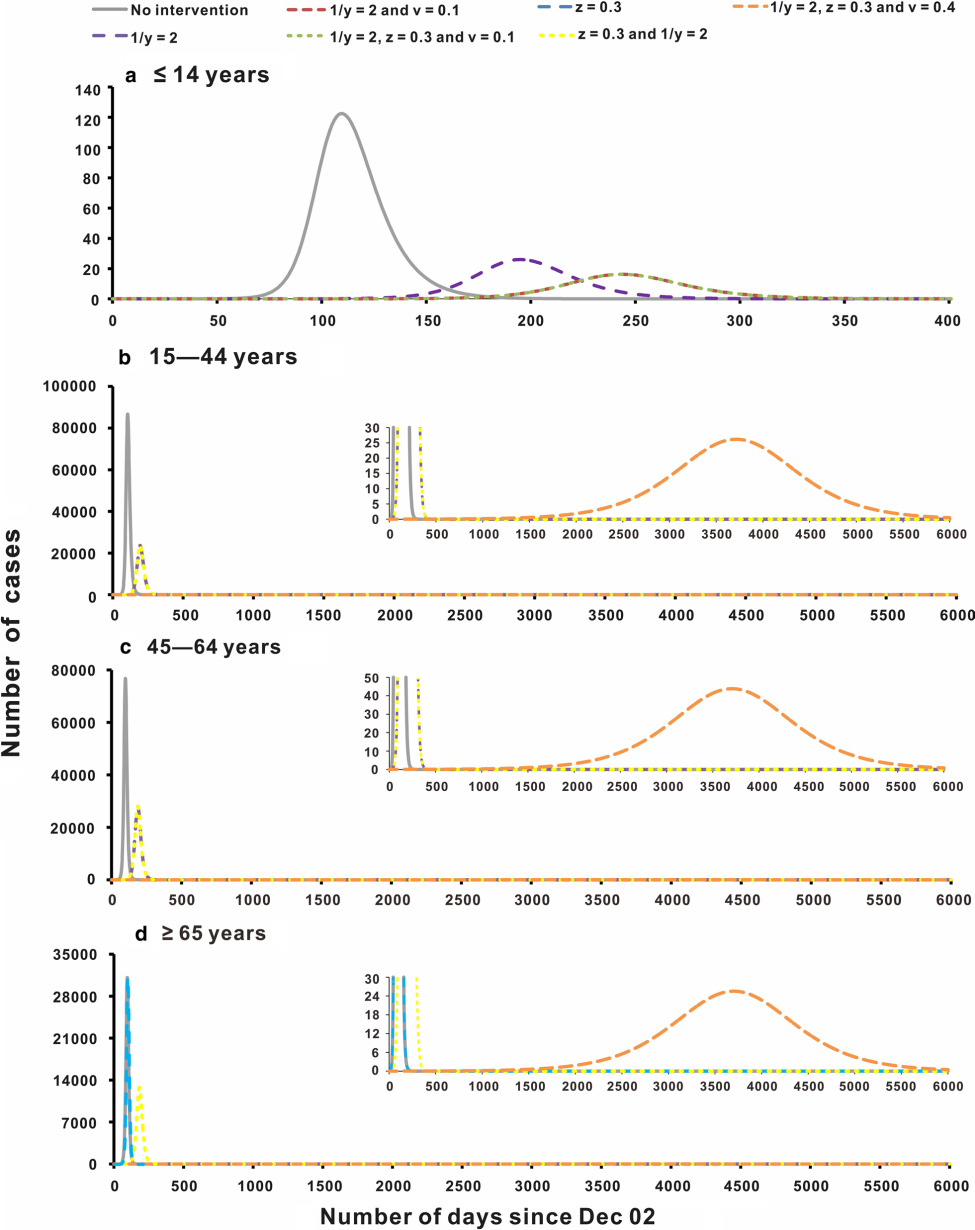
Evaluation of the effectiveness of potential antiviral treatments (with highest *f* reduction in each age group). **a** ≤ 14 years; **b** 15–44 years; **c** 45–64 years; **d** ≥ 65 years

Comparing the simulation results of combination and single medications, all four age groups showed better results with the combination medication. When 1/*γ* = 2, and v = 0.6 or 1/*γ* = 2, v = 0.6, and *z* = 0.3, age group 1 had the highest TAR reduction rate. However, When 1/*γ* = 2 and v = 0.4 or 1/*γ* = 2, v = 0.4, and *z* = 0.3, age group 2–4 had the highest TAR reduction rates. The TAR reduction rate of age group 2 was the highest among the 4 age groups considered (98.48%, from 56.01 to 0.85%) (Fig. 3b), whereas the absolute TAR reduction value of age group 3 was highest among the 4 age groups (0.6070, from 0.6292 to 0.0222) (Fig. 3c). When 1/*γ* = 2, *z* = 0.3, and v = 0.1, age group 1 had the highest *f* reduction rate. However, when v = 0.4, *z* = 0.3, and 1/*γ* = 2, age group 2–4 had the highest *f* reduction rate. The *f* reduction rate of age group 1 was the highest among all 4 age groups (83.08%, from 0.71 to 0.12%) (Fig. 4a). whereas the absolute *f* reduction value of age group 4 was the highest (0.1451, from 0.2938 to 0.1487) (Fig. 4d). Details are shown in Additional file 8: Table S6, Additional file 9: Table S7, Additional file 10: Table S8, Additional file 11: Table S9.

### The results of the sensitivity analysis

**Fig. 5:**
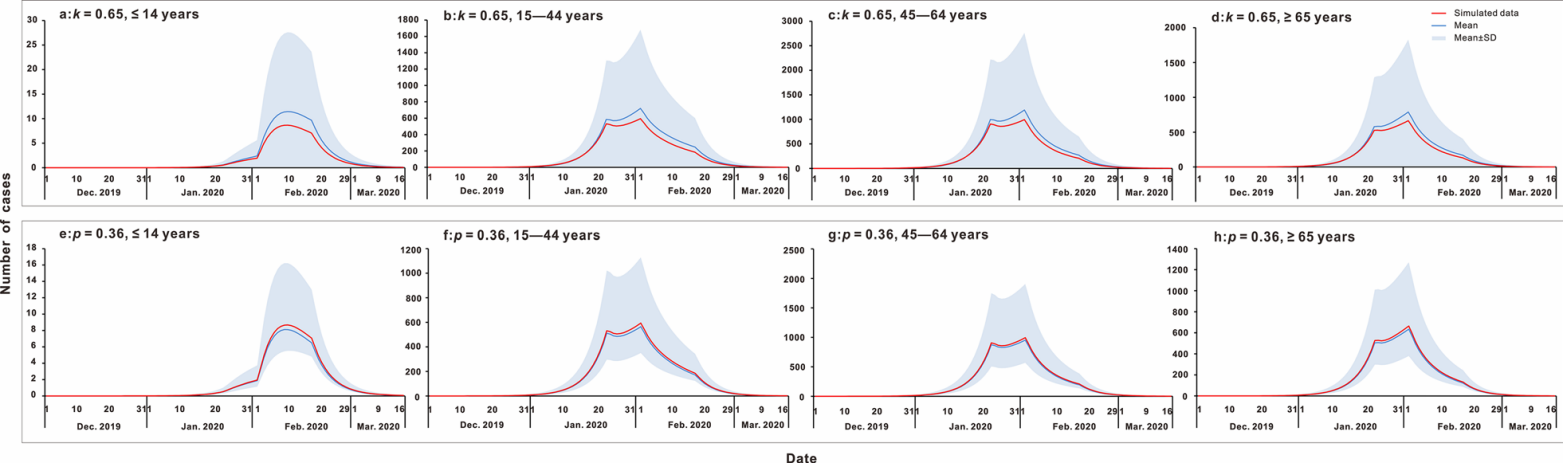
The results of sensitivity analysis. **a** *κ* = 0.65, ≤ 14 years; **b**: *κ* = 0.65, 15–44 years; **c** *κ* = 0.65, 45–64 years; **d** *κ* = 0.65, ≥ 65 years; **e** *p* = 0.36, ≤ 14 years; **f** *p* = 0.36, 15–44 years; **g** *p* = 0.36, 45–64 years; **h** *p* = 0.36, ≥ 65 years

In this study, we found that parameters *k* and *p* used in the model were included in the range of simulated mean ± SD values (Fig. 5). These two parameters were not sensitive to the model.

## Discussion

This study was based on our existing age-specific transmissibility model of SARS-CoV-2, which is the first model to quantify the transmissibility of COVID-19 within and between different age groups [39]. We pursued an innovative approach by incorporating multiple factors, such as the ratio of severely ill patients (*q*) and the fatality rate of severely ill patients (*f*_*c*_) of each age group, to build an antiviral treatments evaluation model for a set of age groups. The model was used to assess intervention efficacy of different antiviral treatments by changing parameters as needed. However, most current COVID-19 pharmacological therapies remain at the clinical efficacy evaluation stage, and the reliability of antiviral treatments require further exploration. The focus of this study was to evaluate the effectiveness of antiviral treatments in different age groups from a public health perspective, using hypothetical or real-life antiviral treatments.

Parameters of this study were as follows: *β* and disease natural history parameters. *β* parameters were obtained from the results of curve fitting. The date separating stages 1 and 2 was 23 January 2020, as Wuhan declared a lockdown on 22 January 2020. Thus, we can approximately regard the first stage as the non-intervention stage. It is universally accepted that *β* parameters of stage 1 represent the transmissibility of the disease in its natural state [31]. This is also the possible reason why the number of simulated cases exceeded the actual number of cases in Wuhan. Under this premise, we excluded the efficacy of other possible interventions when evaluating the efficacy of antiviral treatments. Moreover, results of *R*^*2*^ calculations showed that the model fitted well.

There are two sources of disease natural history parameters: the ratio of severely ill patients (*q*) and the fatality rate of severely ill patients (*f*_*c*_), which were calculated using real data. Remaining parameters (*κ*, *p*, *ω*, *ω′*, *γ*, and *γ′*) were quoted from prior studies. Sensitivity analyses were performed for parameters of *k* and *p*, and other values were based on published findings. Median values of published findings were calculated.

Some studies have reported variations in COVID-19 prevalence between different age groups [35, 36, 39], akin to our study findings. Without intervention, groups 1 and 4 had the lowest and highest TAR values, respectively; these were similar to findings of a study conducted in Hungary [37]. Another three-age-group study using a generalised linear mixed model revealed that people aged ≥ 65 years had a higher risk of becoming infected with SARS-CoV-2 than those aged 15–64 years [70]. It was speculated that reasons for these age-based differences may be due to increased rates of underlying diseases observed in adults aged 65 years [38] that predisposed them to COVID-19 infection due to their low level of immunity. Differing fatality rates observed in severely ill patients did not have an impact on the epidemic. This may be due to the fact that most severely ill patients are in the intensive care units and reducing the fatality rate will not affect other populations.

When antiviral treatments reduce *β* or *γ* and *γ’*, each age group had a different degree of control. This may have been due to differences in disease transmissibility observed for each age group. For example, the age group 2 population was the largest group, which may suggest that the decrease in *β* had the greatest impact on the TAR in the age group. Moreover, we hypothesised that different age groups had different sensitivities to different antiviral treatments. However, the specific antiviral treatment sensitivity of each age group requires further pharmacological exploration. Furthermore, differences in autoimmunity may have an effect on the effectiveness of potential antiviral treatments.

Some parameters were not included in the study, such as data associated with non-severe cases and that of immunity acquisition over time. Future scenario predictions may be inaccurate and unpredictable, and current calculation methods may overestimate or underestimate the effectiveness of potential antiviral treatments throughout the population considered.

## Conclusions

Antiviral treatments are more effective for COVID-19 transmission control than for mortality reduction. Overall, antiviral treatments were most effective when used to treat younger age groups, while older age groups showed higher disease prevalence and mortality. Therefore, older age groups require more attention with respect to the use of antiviral treatments in clinical practice.

## Supplementary Information

**Additional file 1: Fig. S1.** Curve fitting of the age-specific SEIAR model to the reported data in Wuhan City. a: ≤ 14 years; b: 15–44 years; c: 45–64 years; d: ≥ 65 years.

**Additional file 2: Fig. S2.** The value of 4 age groups’ transmission (β) in 4 stage. a: ≤ 14 years; b:15–44 years; c: 45–64 years; d: ≥ 65 years; stage 1: December 2, 2019 to January 23, 2020; stage 2: January 24 to February 2, 2020; stage 3: February 3 to February 18, 2020; stage 4: February 19, 2020 to March 16, 2020.

**Additional file 3: Table S1.** The results of goodness of fit in four stages of four age groups.

**Additional file 4: Table S2.** The effectiveness of potential antiviral treatments in group 1 (ages ≤ 14 years).

**Additional file 5: Table S3.** The effectiveness of potential antiviral treatments in group 2 (ages 15–44 years).

**Additional file 6: Table S4.** The effectiveness of potential antiviral treatments in group 3 (ages 45–64 years).

**Additional file 7: Table S5.** The effectiveness of potential antiviral treatments in group 4 (ages ≥ 65 years).

**Additional file 8: Table S6.** The reduction rate of total attack rate (TAR).

**Additional file 9: Table S7.** The absolute reduction value of total attack rate (TAR).

**Additional file 10: Table S8.** The reduction rate of case fatality rate (*f*).

**Additional file 11: Table S9.** The absolute reduction value of case fatality rate (*f*).

## Data Availability

The datasets used and analysed during the current study are available from Miss Shengnan Lin (shengnanlin0228@163.com) on reasonable request.

## Abbreviations

*A*: Asymptomatic
CNC: Cumulative number of cases
COVID-19: Novel coronavirus disease 2019
*E*: Exposed
*f*: Case fatality rate
*f*_*c*_: The fatality rate of severely ill patients
*I*: Symptomatic
NPC: Number of peak cases
OD: Outbreak duration
PD: Peak date
*R*: Recovered/removed
*S*: Susceptible
SARS-CoV-2: Severe acute respiratory syndrome coronavirus 2
SEIAR: Susceptible–exposed–symptomatic–asymptomatic-recovered/removed
TAR: Total attack rate
WHO: World Health Organisation
