# Supplementary figures and images for: Effectiveness of potential antiviral treatments in COVID-19 transmission control: a modelling study
Source: Infect Dis Poverty. 2021 Apr 19;10:53. doi: 10.1186/s40249-021-00835-2 (PMC8054260; doi:10.1186/s40249-021-00835-2)

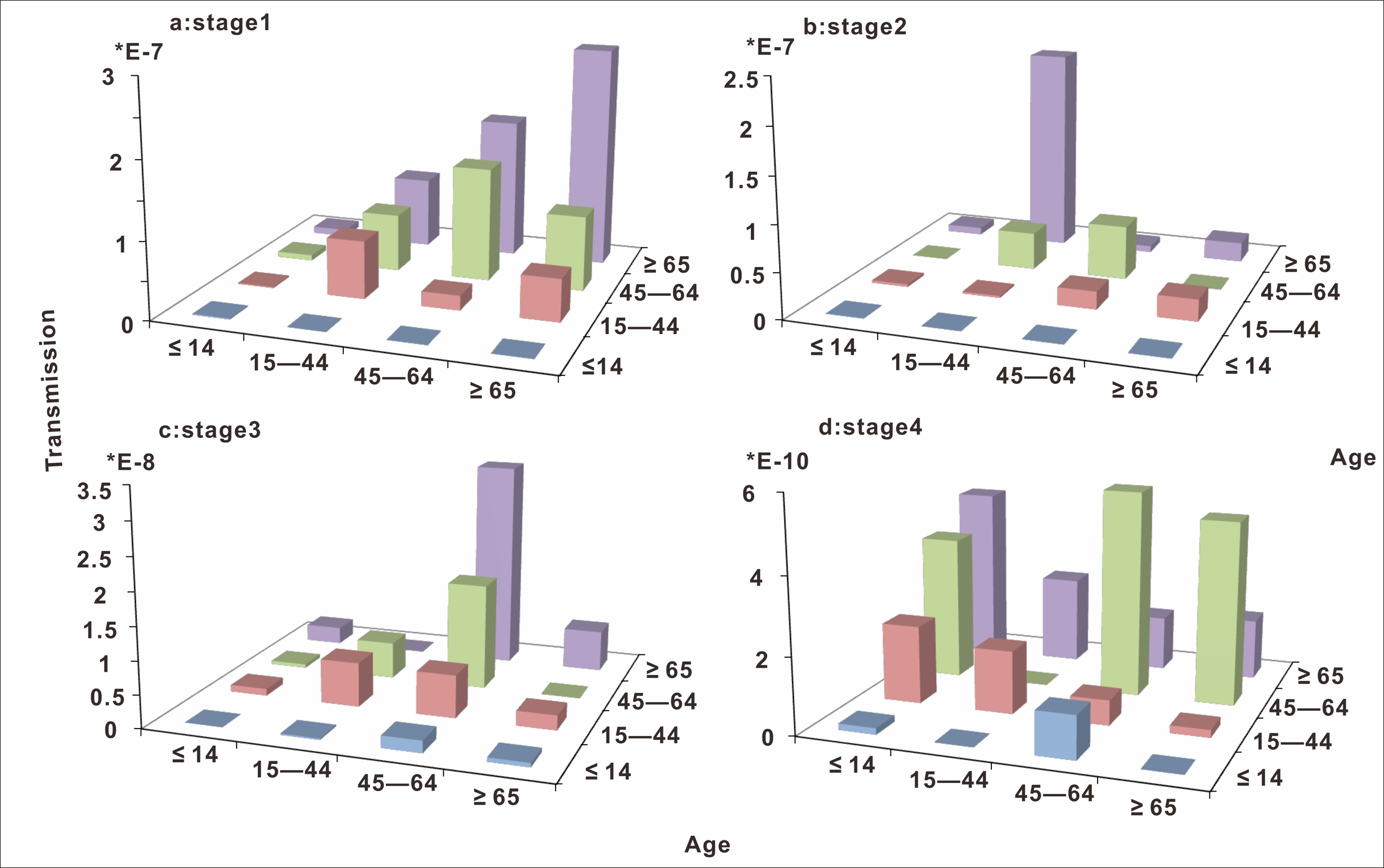

Supplement: Supplementary file 2 — Additional file 2: Fig. S2. The value of 4 age groups’ transmission (β) in 4 stage. a: ≤ 14 years; b:15–44 years; c: 45–64 years; d: ≥ 65 years; stage 1: December 2, 2019 to January 23, 2020; stage 2: January 24 to February 2, 2020; stage 3: February 3 to February 18, 2020; stage 4: February 19, 2020 to March 16, 2020. [file 40249_2021_835_MOESM2_ESM.jpg]
