# Supplementary material for: Effectiveness of potential antiviral treatments in COVID-19 transmission control: a modelling study
Source: Infect Dis Poverty. 2021 Apr 19;10:53. doi: 10.1186/s40249-021-00835-2 (PMC8054260; doi:10.1186/s40249-021-00835-2)
Supplement: Supplementary file 3 — Additional file 3: Table S1. The results of goodness of fit in four stages of four age groups. [file 40249_2021_835_MOESM3_ESM.docx]

**Additional Table 1 The results of goodness of fit in four stages of four age groups.**

|  | stage 1 | stage 2 | stage 3 | stage 4 |
| --- | --- | --- | --- | --- |
| age1 | 0.673*** | 0.526** | 0.154 | 0.853*** |
| age2 | 0.976*** | 0.691*** | 0.837*** | 0.913*** |
| age3 | 0.987*** | 0.806*** | 0.884*** | 0.899*** |
| age4 | 0.988*** | 0.787*** | 0.887*** | 0.812*** |

age 1: ≤ 14 years; age 2: 15–44 years; age 3: 45–64 years; age 4: ≥ 65 years.

stage 1: December 2, 2019 to January 23, 2020; stage 2: January 24 to February 2, 2020; stage 3: February 3 to February 18, 2020; stage 4: February 19, 2020 to March 16, 2020.

*: *p*<0.05; **: *p*<0.01; ***: *p*<0.001.
