# Supplementary material for: Effectiveness of potential antiviral treatments in COVID-19 transmission control: a modelling study
Source: Infect Dis Poverty. 2021 Apr 19;10:53. doi: 10.1186/s40249-021-00835-2 (PMC8054260; doi:10.1186/s40249-021-00835-2)
Supplement: Supplementary file 4 — Additional file 4: Table S2. The effectiveness of potential antiviral treatments in group 1 (ages ≤ 14 years). [file 40249_2021_835_MOESM4_ESM.docx]

**Additional Table 2 The effectiveness of potential antiviral treatments in group 1 (ages ≤ 14 years)**

| model | v | z | γ | γ' | OD | CNC | TAR | PD | NPC | *f* |
| --- | --- | --- | --- | --- | --- | --- | --- | --- | --- | --- |
| 1 | 0 | 0 | 0.2 | 0.1 | 183 | 4656 | 0.0037 | 110 | 122 | 0.00707689 |
| 2 | 0.1 | 0 | 0.2 | 0.1 | 198 | 4101 | 0.0033 | 122 | 100 | 0.00704706 |
| 3 | 0.2 | 0 | 0.2 | 0.1 | 219 | 3530 | 0.0028 | 139 | 77 | 0.00701133 |
| 4 | 0.3 | 0 | 0.2 | 0.1 | 249 | 2936 | 0.0023 | 164 | 56 | 0.00693120 |
| 5 | 0.4 | 0 | 0.2 | 0.1 | 295 | 2309 | 0.0018 | 203 | 37 | 0.00679948 |
| 6 | 0.5 | 0 | 0.2 | 0.1 | 378 | 1637 | 0.0013 | 276 | 20 | 0.00647526 |
| 7 | 0.6 | 0 | 0.2 | 0.1 | 583 | 895 | 0.0007 | 464 | 7 | - |
| 8 | 0.7 | 0 | 0.2 | 0.1 | - | - | - | - | - | - |
| 9 | 0.8 | 0 | 0.2 | 0.1 | - | - | - | - | - | - |
| 10 | 0 | 0.3 | 0.2 | 0.1 | 183 | 4656 | 0.0037 | 110 | 122 | 0.00495382 |
| 11 | 0.1 | 0.3 | 0.2 | 0.1 | 198 | 4101 | 0.0033 | 122 | 100 | 0.00493294 |
| 12 | 0.2 | 0.3 | 0.2 | 0.1 | 219 | 3530 | 0.0028 | 139 | 77 | 0.00490793 |
| 13 | 0.3 | 0.3 | 0.2 | 0.1 | 249 | 2936 | 0.0023 | 164 | 56 | 0.00485184 |
| 14 | 0.4 | 0.3 | 0.2 | 0.1 | 295 | 2309 | 0.0018 | 203 | 37 | 0.00475964 |
| 15 | 0.5 | 0.3 | 0.2 | 0.1 | 378 | 1637 | 0.0013 | 276 | 20 | 0.00453268 |
| 16 | 0.6 | 0.3 | 0.2 | 0.1 | 583 | 895 | 0.0007 | 464 | 7 | - |
| 17 | 0.7 | 0.3 | 0.2 | 0.1 | - | - | - | - | - | - |
| 18 | 0.8 | 0.3 | 0.2 | 0.1 | - | - | - | - | - | - |
| 19 | 0 | 0 | 0.25 | 0.125 | 188 | 3762 | 0.0030 | 119 | 96 | 0.00578150 |
| 20 | 0.1 | 0 | 0.25 | 0.125 | 207 | 3273 | 0.0026 | 134 | 76 | 0.00572869 |
| 21 | 0.2 | 0 | 0.25 | 0.125 | 232 | 2766 | 0.0022 | 155 | 57 | 0.00567607 |
| 22 | 0.3 | 0 | 0.25 | 0.125 | 270 | 2234 | 0.0018 | 187 | 39 | 0.00555058 |
| 23 | 0.4 | 0 | 0.25 | 0.125 | 334 | 1670 | 0.0013 | 242 | 23 | 0.00529940 |
| 24 | 0.5 | 0 | 0.25 | 0.125 | 463 | 1058 | 0.0008 | 358 | 10 | 0.00415879 |
| 25 | 0.6 | 0 | 0.25 | 0.125 | 904 | 349 | 0.0003 | 806 | 2 | - |
| 26 | 0.7 | 0 | 0.25 | 0.125 | - | - | - | - | - | - |
| 27 | 0.8 | 0 | 0.25 | 0.125 | - | - | - | - | - | - |
| 28 | 0 | 0.3 | 0.25 | 0.125 | 188 | 3762 | 0.0030 | 119 | 96 | 0.00404705 |
| 29 | 0.1 | 0.3 | 0.25 | 0.125 | 207 | 3273 | 0.0026 | 134 | 76 | 0.00401008 |
| 30 | 0.2 | 0.3 | 0.25 | 0.125 | 232 | 2766 | 0.0022 | 155 | 57 | 0.00397325 |
| 31 | 0.3 | 0.3 | 0.25 | 0.125 | 270 | 2234 | 0.0018 | 187 | 39 | 0.00388541 |
| 32 | 0.4 | 0.3 | 0.25 | 0.125 | 334 | 1670 | 0.0013 | 242 | 23 | 0.00370958 |
| 33 | 0.5 | 0.3 | 0.25 | 0.125 | 463 | 1058 | 0.0008 | 358 | 10 | 0.00291115 |
| 34 | 0.6 | 0.3 | 0.25 | 0.125 | 904 | 349 | 0.0003 | 806 | 2 | - |
| 35 | 0.7 | 0.3 | 0.25 | 0.125 | - | - | - | - | - | - |
| 36 | 0.8 | 0.3 | 0.25 | 0.125 | - | - | - | - | - | - |
| 37 | 0 | 0 | 0.33 | 0.167 | 206 | 2747 | 0.0022 | 137 | 64 | 0.00435020 |
| 38 | 0.1 | 0 | 0.33 | 0.167 | 231 | 2325 | 0.0019 | 158 | 48 | 0.00427957 |
| 39 | 0.2 | 0 | 0.33 | 0.167 | 269 | 1886 | 0.0015 | 190 | 33 | 0.00410923 |
| 40 | 0.3 | 0 | 0.33 | 0.167 | 329 | 1420 | 0.0011 | 243 | 20 | 0.00376761 |
| 41 | 0.4 | 0 | 0.33 | 0.167 | 449 | 921 | 0.0007 | 351 | 9 | - |
| 42 | 0.5 | 0 | 0.33 | 0.167 | 803 | 353 | 0.0003 | 708 | 2 | - |
| 43 | 0.6 | 0 | 0.33 | 0.167 | - | - | - | - | - | - |
| 44 | 0.7 | 0 | 0.33 | 0.167 | - | - | - | - | - | - |
| 45 | 0.8 | 0 | 0.33 | 0.167 | - | - | - | - | - | - |
| 46 | 0 | 0.3 | 0.33 | 0.167 | 206 | 2747 | 0.0022 | 137 | 64 | 0.00304514 |
| 47 | 0.1 | 0.3 | 0.33 | 0.167 | 231 | 2325 | 0.0019 | 158 | 48 | 0.00299570 |
| 48 | 0.2 | 0.3 | 0.33 | 0.167 | 269 | 1886 | 0.0015 | 190 | 33 | 0.00287646 |
| 49 | 0.3 | 0.3 | 0.33 | 0.167 | 329 | 1420 | 0.0011 | 243 | 20 | 0.00263732 |
| 50 | 0.4 | 0.3 | 0.33 | 0.167 | 449 | 921 | 0.0007 | 351 | 9 | - |
| 51 | 0.5 | 0.3 | 0.33 | 0.167 | 803 | 353 | 0.0003 | 708 | 2 | - |
| 52 | 0.6 | 0.3 | 0.33 | 0.167 | - | - | - | - | - | - |
| 53 | 0.7 | 0.3 | 0.33 | 0.167 | - | - | - | - | - | - |
| 54 | 0.8 | 0.3 | 0.33 | 0.167 | - | - | - | - | - | - |
| 55 | 0 | 0 | 0.5 | 0.25 | 269 | 1515 | 0.0012 | 195 | 26 | 0.00250825 |
| 56 | 0.1 | 0 | 0.5 | 0.25 | 325 | 1169 | 0.0009 | 244 | 16 | 0.00171086 |
| 57 | 0.2 | 0 | 0.5 | 0.25 | 424 | 801 | 0.0006 | 334 | 8 | - |
| 58 | 0.3 | 0 | 0.5 | 0.25 | 660 | 397 | 0.0003 | 566 | 3 | - |
| 59 | 0.4 | 0 | 0.5 | 0.25 | - | - | - | - | - | - |
| 60 | 0.5 | 0 | 0.5 | 0.25 | - | - | - | - | - | - |
| 61 | 0.6 | 0 | 0.5 | 0.25 | - | - | - | - | - | - |
| 62 | 0.7 | 0 | 0.5 | 0.25 | - | - | - | - | - | - |
| 63 | 0.8 | 0 | 0.5 | 0.25 | - | - | - | - | - | - |
| 64 | 0 | 0.3 | 0.5 | 0.25 | 269 | 1515 | 0.0012 | 195 | 26 | 0.00175578 |
| 65 | 0.1 | 0.3 | 0.5 | 0.25 | 325 | 1169 | 0.0009 | 244 | 16 | 0.00119760 |
| 66 | 0.2 | 0.3 | 0.5 | 0.25 | 424 | 801 | 0.0006 | 334 | 8 | - |
| 67 | 0.3 | 0.3 | 0.5 | 0.25 | 660 | 397 | 0.0003 | 566 | 3 | - |
| 68 | 0.4 | 0.3 | 0.5 | 0.25 | - | - | - | - | - | - |
| 69 | 0.5 | 0.3 | 0.5 | 0.25 | - | - | - | - | - | - |
| 70 | 0.6 | 0.3 | 0.5 | 0.25 | - | - | - | - | - | - |
| 71 | 0.7 | 0.3 | 0.5 | 0.25 | - | - | - | - | - | - |
| 72 | 0.8 | 0.3 | 0.5 | 0.25 | - | - | - | - | - | - |

OD=outbreak duration. CNC= cumulative number of cases. TAR= total attack rate.

PD= peak date. NPC= number of peak cases. *f*= case fatality rate.

-= has been controlled
