# Supplementary material for: Effectiveness of potential antiviral treatments in COVID-19 transmission control: a modelling study
Source: Infect Dis Poverty. 2021 Apr 19;10:53. doi: 10.1186/s40249-021-00835-2 (PMC8054260; doi:10.1186/s40249-021-00835-2)
Supplement: Supplementary file 5 — Additional file 5: Table S3. The effectiveness of potential antiviral treatments in group 2 (ages 15–44 years). [file 40249_2021_835_MOESM5_ESM.docx]

**Additional Table 3 The effectiveness of potential antiviral treatments in group 2 (ages 15–44 years)**

| model | v | z | γ | γ' | OD | CNC | TAR | PD | NPC | *f* |
| --- | --- | --- | --- | --- | --- | --- | --- | --- | --- | --- |
| 1 | 0 | 0 | 0.2 | 0.1 | 260 | 2918525 | 0.5601 | 106 | 86680 | 0.01967986 |
| 2 | 0.1 | 0 | 0.2 | 0.1 | 284 | 2786531 | 0.5348 | 118 | 73931 | 0.01967988 |
| 3 | 0.2 | 0 | 0.2 | 0.1 | 317 | 2607070 | 0.5003 | 136 | 60486 | 0.01967983 |
| 4 | 0.3 | 0 | 0.2 | 0.1 | 362 | 2360752 | 0.4530 | 161 | 46445 | 0.01967977 |
| 5 | 0.4 | 0 | 0.2 | 0.1 | 432 | 2020586 | 0.3878 | 201 | 32146 | 0.01967971 |
| 6 | 0.5 | 0 | 0.2 | 0.1 | 555 | 1552420 | 0.2979 | 274 | 18308 | 0.01967950 |
| 7 | 0.6 | 0 | 0.2 | 0.1 | 859 | 923778 | 0.1773 | 462 | 6498 | 0.01967865 |
| 8 | 0.7 | 0 | 0.2 | 0.1 | 4262 | 123018 | 0.0236 | 2673 | 121 | 0.01961287 |
| 9 | 0.8 | 0 | 0.2 | 0.1 | - | - | - | - | - | - |
| 10 | 0 | 0.3 | 0.2 | 0.1 | 260 | 2918525 | 0.5601 | 106 | 86680 | 0.01377590 |
| 11 | 0.1 | 0.3 | 0.2 | 0.1 | 284 | 2786531 | 0.5348 | 118 | 73931 | 0.01377592 |
| 12 | 0.2 | 0.3 | 0.2 | 0.1 | 317 | 2607070 | 0.5003 | 136 | 60486 | 0.01377588 |
| 13 | 0.3 | 0.3 | 0.2 | 0.1 | 362 | 2360752 | 0.4530 | 161 | 46445 | 0.01377584 |
| 14 | 0.4 | 0.3 | 0.2 | 0.1 | 432 | 2020586 | 0.3878 | 201 | 32146 | 0.01377580 |
| 15 | 0.5 | 0.3 | 0.2 | 0.1 | 555 | 1552420 | 0.2979 | 274 | 18308 | 0.01377565 |
| 16 | 0.6 | 0.3 | 0.2 | 0.1 | 859 | 923778 | 0.1773 | 462 | 6498 | 0.01377506 |
| 17 | 0.7 | 0.3 | 0.2 | 0.1 | 4262 | 123018 | 0.0236 | 2673 | 121 | 0.01372901 |
| 18 | 0.8 | 0.3 | 0.2 | 0.1 | - | - | - | - | - | - |
| 19 | 0 | 0 | 0.25 | 0.125 | 268 | 2677992 | 0.5139 | 115 | 72866 | 0.01704466 |
| 20 | 0.1 | 0 | 0.25 | 0.125 | 296 | 2499800 | 0.4797 | 131 | 60288 | 0.01704464 |
| 21 | 0.2 | 0 | 0.25 | 0.125 | 335 | 2267134 | 0.4351 | 152 | 47248 | 0.01704460 |
| 22 | 0.3 | 0 | 0.25 | 0.125 | 392 | 1962609 | 0.3766 | 185 | 34050 | 0.01704452 |
| 23 | 0.4 | 0 | 0.25 | 0.125 | 485 | 1565921 | 0.3005 | 240 | 21182 | 0.01704436 |
| 24 | 0.5 | 0 | 0.25 | 0.125 | 675 | 1058432 | 0.2031 | 357 | 9708 | 0.01704386 |
| 25 | 0.6 | 0 | 0.25 | 0.125 | 1396 | 431878 | 0.0829 | 806 | 1673 | 0.01703952 |
| 26 | 0.7 | 0 | 0.25 | 0.125 | - | - | - | - | - | - |
| 27 | 0.8 | 0 | 0.25 | 0.125 | - | - | - | - | - | - |
| 28 | 0 | 0.3 | 0.25 | 0.125 | 268 | 2677992 | 0.5139 | 115 | 72866 | 0.01193127 |
| 29 | 0.1 | 0.3 | 0.25 | 0.125 | 296 | 2499800 | 0.4797 | 131 | 60288 | 0.01193124 |
| 30 | 0.2 | 0.3 | 0.25 | 0.125 | 335 | 2267134 | 0.4351 | 152 | 47248 | 0.01193122 |
| 31 | 0.3 | 0.3 | 0.25 | 0.125 | 392 | 1962609 | 0.3766 | 185 | 34050 | 0.01193116 |
| 32 | 0.4 | 0.3 | 0.25 | 0.125 | 485 | 1565921 | 0.3005 | 240 | 21182 | 0.01193105 |
| 33 | 0.5 | 0.3 | 0.25 | 0.125 | 675 | 1058432 | 0.2031 | 357 | 9708 | 0.01193070 |
| 34 | 0.6 | 0.3 | 0.25 | 0.125 | 1396 | 431878 | 0.0829 | 806 | 1673 | 0.01192766 |
| 35 | 0.7 | 0.3 | 0.25 | 0.125 | - | - | - | - | - | - |
| 36 | 0.8 | 0.3 | 0.25 | 0.125 | - | - | - | - | - | - |
| 37 | 0 | 0 | 0.33 | 0.167 | 294 | 2242207 | 0.4303 | 135 | 52671 | 0.01393476 |
| 38 | 0.1 | 0 | 0.33 | 0.167 | 332 | 2003996 | 0.3846 | 156 | 40959 | 0.01393469 |
| 39 | 0.2 | 0 | 0.33 | 0.167 | 387 | 1710640 | 0.3283 | 189 | 29348 | 0.01393461 |
| 40 | 0.3 | 0 | 0.33 | 0.167 | 475 | 1352579 | 0.2596 | 242 | 18301 | 0.01393439 |
| 41 | 0.4 | 0 | 0.33 | 0.167 | 652 | 923021 | 0.1771 | 350 | 8657 | 0.01393377 |
| 42 | 0.5 | 0 | 0.33 | 0.167 | 1230 | 420181 | 0.0806 | 708 | 1854 | 0.01392966 |
| 43 | 0.6 | 0 | 0.33 | 0.167 | - | - | - | - | - | - |
| 44 | 0.7 | 0 | 0.33 | 0.167 | - | - | - | - | - | - |
| 45 | 0.8 | 0 | 0.33 | 0.167 | - | - | - | - | - | - |
| 46 | 0 | 0.3 | 0.33 | 0.167 | 294 | 2242207 | 0.4303 | 135 | 52671 | 0.00975433 |
| 47 | 0.1 | 0.3 | 0.33 | 0.167 | 332 | 2003996 | 0.3846 | 156 | 40959 | 0.00975428 |
| 48 | 0.2 | 0.3 | 0.33 | 0.167 | 387 | 1710640 | 0.3283 | 189 | 29348 | 0.00975422 |
| 49 | 0.3 | 0.3 | 0.33 | 0.167 | 475 | 1352579 | 0.2596 | 242 | 18301 | 0.00975407 |
| 50 | 0.4 | 0.3 | 0.33 | 0.167 | 652 | 923021 | 0.1771 | 350 | 8657 | 0.00975364 |
| 51 | 0.5 | 0.3 | 0.33 | 0.167 | 1230 | 420181 | 0.0806 | 708 | 1854 | 0.00975076 |
| 52 | 0.6 | 0.3 | 0.33 | 0.167 | - | - | - | - | - | - |
| 53 | 0.7 | 0.3 | 0.33 | 0.167 | - | - | - | - | - | - |
| 54 | 0.8 | 0.3 | 0.33 | 0.167 | - | - | - | - | - | - |
| 55 | 0 | 0 | 0.5 | 0.25 | 387 | 1408563 | 0.2703 | 194 | 23629 | 0.01020900 |
| 56 | 0.1 | 0 | 0.5 | 0.25 | 467 | 1122058 | 0.2153 | 243 | 15157 | 0.01020874 |
| 57 | 0.2 | 0 | 0.5 | 0.25 | 615 | 799577 | 0.1534 | 333 | 7852 | 0.01020809 |
| 58 | 0.3 | 0 | 0.5 | 0.25 | 993 | 440892 | 0.0846 | 565 | 2452 | 0.01020515 |
| 59 | 0.4 | 0 | 0.5 | 0.25 | 5707 | 44487 | 0.0085 | 3713 | 26 | 0.00978338 |
| 60 | 0.5 | 0 | 0.5 | 0.25 | - | - | - | - | - | - |
| 61 | 0.6 | 0 | 0.5 | 0.25 | - | - | - | - | - | - |
| 62 | 0.7 | 0 | 0.5 | 0.25 | - | - | - | - | - | - |
| 63 | 0.8 | 0 | 0.5 | 0.25 | - | - | - | - | - | - |
| 64 | 0 | 0.3 | 0.5 | 0.25 | 387 | 1408563 | 0.2703 | 194 | 23629 | 0.00714630 |
| 65 | 0.1 | 0.3 | 0.5 | 0.25 | 467 | 1122058 | 0.2153 | 243 | 15157 | 0.00714612 |
| 66 | 0.2 | 0.3 | 0.5 | 0.25 | 615 | 799577 | 0.1534 | 333 | 7852 | 0.00714566 |
| 67 | 0.3 | 0.3 | 0.5 | 0.25 | 993 | 440892 | 0.0846 | 565 | 2452 | 0.00714360 |
| 68 | 0.4 | 0.3 | 0.5 | 0.25 | 5707 | 44487 | 0.0085 | 3713 | 26 | 0.00684836 |
| 69 | 0.5 | 0.3 | 0.5 | 0.25 | - | - | - | - | - | - |
| 70 | 0.6 | 0.3 | 0.5 | 0.25 | - | - | - | - | - | - |
| 71 | 0.7 | 0.3 | 0.5 | 0.25 | - | - | - | - | - | - |
| 72 | 0.8 | 0.3 | 0.5 | 0.25 | - | - | - | - | - | - |

OD=outbreak duration. CNC= cumulative number of cases. TAR= total attack rate.

PD= peak date. NPC= number of peak cases. *f*= case fatality rate.

-= has been controlled
