# Supplementary material for: Effectiveness of potential antiviral treatments in COVID-19 transmission control: a modelling study
Source: Infect Dis Poverty. 2021 Apr 19;10:53. doi: 10.1186/s40249-021-00835-2 (PMC8054260; doi:10.1186/s40249-021-00835-2)
Supplement: Supplementary file 6 — Additional file 6: Table S4. The effectiveness of potential antiviral treatments in group 3 (ages 45–64 years). [file 40249_2021_835_MOESM6_ESM.docx]

**Additional Table 4 The effectiveness of potential antiviral treatments in** **group 3 (ages 45–64 years)**

| model | v | z | γ | γ' | OD | CNC | TAR | PD | NPC | *f* |
| --- | --- | --- | --- | --- | --- | --- | --- | --- | --- | --- |
| 1 | 0 | 0 | 0.2 | 0.1 | 230 | 2123203 | 0.6292 | 99 | 76719 | 0.09862580 |
| 2 | 0.1 | 0 | 0.2 | 0.1 | 256 | 2099949 | 0.6223 | 112 | 67278 | 0.09862559 |
| 3 | 0.2 | 0 | 0.2 | 0.1 | 290 | 2060314 | 0.6106 | 129 | 56941 | 0.09862557 |
| 4 | 0.3 | 0 | 0.2 | 0.1 | 337 | 1990991 | 0.5900 | 153 | 45738 | 0.09862555 |
| 5 | 0.4 | 0 | 0.2 | 0.1 | 410 | 1865662 | 0.5529 | 192 | 33644 | 0.09862519 |
| 6 | 0.5 | 0 | 0.2 | 0.1 | 538 | 1629563 | 0.4829 | 265 | 20901 | 0.09862480 |
| 7 | 0.6 | 0 | 0.2 | 0.1 | 854 | 1163334 | 0.3448 | 452 | 8470 | 0.09862319 |
| 8 | 0.7 | 0 | 0.2 | 0.1 | 4375 | 198473 | 0.0588 | 2662 | 195 | 0.09851808 |
| 9 | 0.8 | 0 | 0.2 | 0.1 | - | - | - | - | - | - |
| 10 | 0 | 0.3 | 0.2 | 0.1 | 230 | 2123203 | 0.6292 | 99 | 76719 | 0.06903806 |
| 11 | 0.1 | 0.3 | 0.2 | 0.1 | 256 | 2099949 | 0.6223 | 112 | 67278 | 0.06903791 |
| 12 | 0.2 | 0.3 | 0.2 | 0.1 | 290 | 2060314 | 0.6106 | 129 | 56941 | 0.06903790 |
| 13 | 0.3 | 0.3 | 0.2 | 0.1 | 337 | 1990991 | 0.5900 | 153 | 45738 | 0.06903789 |
| 14 | 0.4 | 0.3 | 0.2 | 0.1 | 410 | 1865662 | 0.5529 | 192 | 33644 | 0.06903763 |
| 15 | 0.5 | 0.3 | 0.2 | 0.1 | 538 | 1629563 | 0.4829 | 265 | 20901 | 0.06903736 |
| 16 | 0.6 | 0.3 | 0.2 | 0.1 | 854 | 1163334 | 0.3448 | 452 | 8470 | 0.06903623 |
| 17 | 0.7 | 0.3 | 0.2 | 0.1 | 4375 | 198473 | 0.0588 | 2662 | 195 | 0.06896266 |
| 18 | 0.8 | 0.3 | 0.2 | 0.1 | - | - | - | - | - | - |
| 19 | 0 | 0 | 0.25 | 0.125 | 244 | 2078315 | 0.6159 | 109 | 67562 | 0.08849403 |
| 20 | 0.1 | 0 | 0.25 | 0.125 | 274 | 2033958 | 0.6028 | 124 | 57810 | 0.08849389 |
| 21 | 0.2 | 0 | 0.25 | 0.125 | 314 | 1962310 | 0.5815 | 145 | 47287 | 0.08849384 |
| 22 | 0.3 | 0 | 0.25 | 0.125 | 373 | 1843657 | 0.5464 | 177 | 36014 | 0.08849361 |
| 23 | 0.4 | 0 | 0.25 | 0.125 | 470 | 1641277 | 0.4864 | 232 | 24148 | 0.08849322 |
| 24 | 0.5 | 0 | 0.25 | 0.125 | 668 | 1284574 | 0.3807 | 348 | 12304 | 0.08849217 |
| 25 | 0.6 | 0 | 0.25 | 0.125 | 1412 | 634775 | 0.1881 | 797 | 2478 | 0.08848470 |
| 26 | 0.7 | 0 | 0.25 | 0.125 | - | - | - | - | - | - |
| 27 | 0.8 | 0 | 0.25 | 0.125 | - | - | - | - | - | - |
| 28 | 0 | 0.3 | 0.25 | 0.125 | 244 | 2078315 | 0.6159 | 109 | 67562 | 0.06194582 |
| 29 | 0.1 | 0.3 | 0.25 | 0.125 | 274 | 2033958 | 0.6028 | 124 | 57810 | 0.06194572 |
| 30 | 0.2 | 0.3 | 0.25 | 0.125 | 314 | 1962310 | 0.5815 | 145 | 47287 | 0.06194569 |
| 31 | 0.3 | 0.3 | 0.25 | 0.125 | 373 | 1843657 | 0.5464 | 177 | 36014 | 0.06194552 |
| 32 | 0.4 | 0.3 | 0.25 | 0.125 | 470 | 1641277 | 0.4864 | 232 | 24148 | 0.06194526 |
| 33 | 0.5 | 0.3 | 0.25 | 0.125 | 668 | 1284574 | 0.3807 | 348 | 12304 | 0.06194452 |
| 34 | 0.6 | 0.3 | 0.25 | 0.125 | 1412 | 634775 | 0.1881 | 797 | 2478 | 0.06193929 |
| 35 | 0.7 | 0.3 | 0.25 | 0.125 | - | - | - | - | - | - |
| 36 | 0.8 | 0.3 | 0.25 | 0.125 | - | - | - | - | - | - |
| 37 | 0 | 0 | 0.33 | 0.167 | 276 | 1956970 | 0.5799 | 128 | 52940 | 0.07555726 |
| 38 | 0.1 | 0 | 0.33 | 0.167 | 316 | 1865595 | 0.5529 | 150 | 43047 | 0.07555718 |
| 39 | 0.2 | 0 | 0.33 | 0.167 | 373 | 1726802 | 0.5117 | 181 | 32606 | 0.07555695 |
| 40 | 0.3 | 0 | 0.33 | 0.167 | 465 | 1511771 | 0.4480 | 234 | 21847 | 0.07555658 |
| 41 | 0.4 | 0 | 0.33 | 0.167 | 647 | 1171700 | 0.3472 | 342 | 11366 | 0.07555531 |
| 42 | 0.5 | 0 | 0.33 | 0.167 | 1244 | 622949 | 0.1846 | 700 | 2769 | 0.07554808 |
| 43 | 0.6 | 0 | 0.33 | 0.167 | - | - | - | - | - | - |
| 44 | 0.7 | 0 | 0.33 | 0.167 | - | - | - | - | - | - |
| 45 | 0.8 | 0 | 0.33 | 0.167 | - | - | - | - | - | - |
| 46 | 0 | 0.3 | 0.33 | 0.167 | 276 | 1956970 | 0.5799 | 128 | 52940 | 0.05289008 |
| 47 | 0.1 | 0.3 | 0.33 | 0.167 | 316 | 1865595 | 0.5529 | 150 | 43047 | 0.05289002 |
| 48 | 0.2 | 0.3 | 0.33 | 0.167 | 373 | 1726802 | 0.5117 | 181 | 32606 | 0.05288986 |
| 49 | 0.3 | 0.3 | 0.33 | 0.167 | 465 | 1511771 | 0.4480 | 234 | 21847 | 0.05288960 |
| 50 | 0.4 | 0.3 | 0.33 | 0.167 | 647 | 1171700 | 0.3472 | 342 | 11366 | 0.05288872 |
| 51 | 0.5 | 0.3 | 0.33 | 0.167 | 1244 | 622949 | 0.1846 | 700 | 2769 | 0.05288366 |
| 52 | 0.6 | 0.3 | 0.33 | 0.167 | - | - | - | - | - | - |
| 53 | 0.7 | 0.3 | 0.33 | 0.167 | - | - | - | - | - | - |
| 54 | 0.8 | 0.3 | 0.33 | 0.167 | - | - | - | - | - | - |
| 55 | 0 | 0 | 0.5 | 0.25 | 377 | 1556317 | 0.4612 | 188 | 28024 | 0.05846342 |
| 56 | 0.1 | 0 | 0.5 | 0.25 | 461 | 1349019 | 0.3998 | 236 | 19126 | 0.05846277 |
| 57 | 0.2 | 0 | 0.5 | 0.25 | 614 | 1060067 | 0.3142 | 326 | 10684 | 0.05846147 |
| 58 | 0.3 | 0 | 0.5 | 0.25 | 1005 | 653289 | 0.1936 | 559 | 3663 | 0.05845589 |
| 59 | 0.4 | 0 | 0.5 | 0.25 | 5927 | 74805 | 0.0222 | 3707 | 44 | 0.05773692 |
| 60 | 0.5 | 0 | 0.5 | 0.25 | - | - | - | - | - | - |
| 61 | 0.6 | 0 | 0.5 | 0.25 | - | - | - | - | - | - |
| 62 | 0.7 | 0 | 0.5 | 0.25 | - | - | - | - | - | - |
| 63 | 0.8 | 0 | 0.5 | 0.25 | - | - | - | - | - | - |
| 64 | 0 | 0.3 | 0.5 | 0.25 | 377 | 1556317 | 0.4612 | 188 | 28024 | 0.04092439 |
| 65 | 0.1 | 0.3 | 0.5 | 0.25 | 461 | 1349019 | 0.3998 | 236 | 19126 | 0.04092394 |
| 66 | 0.2 | 0.3 | 0.5 | 0.25 | 614 | 1060067 | 0.3142 | 326 | 10684 | 0.04092303 |
| 67 | 0.3 | 0.3 | 0.5 | 0.25 | 1005 | 653289 | 0.1936 | 559 | 3663 | 0.04091912 |
| 68 | 0.4 | 0.3 | 0.5 | 0.25 | 5927 | 74805 | 0.0222 | 3707 | 44 | 0.04041584 |
| 69 | 0.5 | 0.3 | 0.5 | 0.25 | - | - | - | - | - | - |
| 70 | 0.6 | 0.3 | 0.5 | 0.25 | - | - | - | - | - | - |
| 71 | 0.7 | 0.3 | 0.5 | 0.25 | - | - | - | - | - | - |
| 72 | 0.8 | 0.3 | 0.5 | 0.25 | - | - | - | - | - | - |

OD=outbreak duration. CNC= cumulative number of cases. TAR= total attack rate.

PD= peak date. NPC= number of peak cases. *f*= case fatality rate.

-= has been controlled
