# Supplementary material for: Effectiveness of potential antiviral treatments in COVID-19 transmission control: a modelling study
Source: Infect Dis Poverty. 2021 Apr 19;10:53. doi: 10.1186/s40249-021-00835-2 (PMC8054260; doi:10.1186/s40249-021-00835-2)
Supplement: Supplementary file 7 — Additional file 7: Table S5. The effectiveness of potential antiviral treatments in group 4 (ages ≥ 65 years). [file 40249_2021_835_MOESM7_ESM.docx]

**Additional Table 5 The effectiveness of potential antiviral treatments in** **group 4 (ages ≥ 65 years)**

| model | v | z | γ | γ' | OD | CNC | TAR | PD | NPC | *f* |
| --- | --- | --- | --- | --- | --- | --- | --- | --- | --- | --- |
| 1 | 0 | 0 | 0.2 | 0.1 | 198 | 790655 | 0.6381 | 97 | 31080 | 0.29380626 |
| 2 | 0.1 | 0 | 0.2 | 0.1 | 223 | 788284 | 0.6361 | 109 | 27513 | 0.29380613 |
| 3 | 0.2 | 0 | 0.2 | 0.1 | 257 | 783367 | 0.6322 | 125 | 23594 | 0.29380568 |
| 4 | 0.3 | 0 | 0.2 | 0.1 | 305 | 772828 | 0.6237 | 149 | 19268 | 0.29380546 |
| 5 | 0.4 | 0 | 0.2 | 0.1 | 376 | 749188 | 0.6046 | 187 | 14519 | 0.29380522 |
| 6 | 0.5 | 0 | 0.2 | 0.1 | 503 | 692748 | 0.5590 | 259 | 9376 | 0.29380476 |
| 7 | 0.6 | 0 | 0.2 | 0.1 | 813 | 545765 | 0.4404 | 445 | 4075 | 0.29380053 |
| 8 | 0.7 | 0 | 0.2 | 0.1 | 4216 | 111153 | 0.0897 | 2653 | 110 | 0.29358695 |
| 9 | 0.8 | 0 | 0.2 | 0.1 | - | - | - | - | - | - |
| 10 | 0 | 0.3 | 0.2 | 0.1 | 198 | 790655 | 0.6381 | 97 | 31080 | 0.20566439 |
| 11 | 0.1 | 0.3 | 0.2 | 0.1 | 223 | 788284 | 0.6361 | 109 | 27513 | 0.20566429 |
| 12 | 0.2 | 0.3 | 0.2 | 0.1 | 257 | 783367 | 0.6322 | 125 | 23594 | 0.20566398 |
| 13 | 0.3 | 0.3 | 0.2 | 0.1 | 305 | 772828 | 0.6237 | 149 | 19268 | 0.20566382 |
| 14 | 0.4 | 0.3 | 0.2 | 0.1 | 376 | 749188 | 0.6046 | 187 | 14519 | 0.20566366 |
| 15 | 0.5 | 0.3 | 0.2 | 0.1 | 503 | 692748 | 0.5590 | 259 | 9376 | 0.20566333 |
| 16 | 0.6 | 0.3 | 0.2 | 0.1 | 813 | 545765 | 0.4404 | 445 | 4075 | 0.20566037 |
| 17 | 0.7 | 0.3 | 0.2 | 0.1 | 4216 | 111153 | 0.0897 | 2653 | 110 | 0.20551086 |
| 18 | 0.8 | 0.3 | 0.2 | 0.1 | - | - | - | - | - | - |
| 19 | 0 | 0 | 0.25 | 0.125 | 216 | 785782 | 0.6341 | 106 | 27799 | 0.27667344 |
| 20 | 0.1 | 0 | 0.25 | 0.125 | 246 | 779719 | 0.6292 | 121 | 24065 | 0.27667357 |
| 21 | 0.2 | 0 | 0.25 | 0.125 | 286 | 768054 | 0.6198 | 141 | 20013 | 0.27667355 |
| 22 | 0.3 | 0 | 0.25 | 0.125 | 344 | 744807 | 0.6011 | 173 | 15588 | 0.27667255 |
| 23 | 0.4 | 0 | 0.25 | 0.125 | 439 | 696296 | 0.5619 | 226 | 10807 | 0.27667199 |
| 24 | 0.5 | 0 | 0.25 | 0.125 | 634 | 588744 | 0.4751 | 342 | 5816 | 0.27666979 |
| 25 | 0.6 | 0 | 0.25 | 0.125 | 1359 | 329816 | 0.2662 | 789 | 1296 | 0.27665250 |
| 26 | 0.7 | 0 | 0.25 | 0.125 | - | - | - | - | - | - |
| 27 | 0.8 | 0 | 0.25 | 0.125 | - | - | - | - | - | - |
| 28 | 0 | 0.3 | 0.25 | 0.125 | 216 | 785782 | 0.6341 | 106 | 27799 | 0.19367141 |
| 29 | 0.1 | 0.3 | 0.25 | 0.125 | 246 | 779719 | 0.6292 | 121 | 24065 | 0.19367150 |
| 30 | 0.2 | 0.3 | 0.25 | 0.125 | 286 | 768054 | 0.6198 | 141 | 20013 | 0.19367148 |
| 31 | 0.3 | 0.3 | 0.25 | 0.125 | 344 | 744807 | 0.6011 | 173 | 15588 | 0.19367078 |
| 32 | 0.4 | 0.3 | 0.25 | 0.125 | 439 | 696296 | 0.5619 | 226 | 10807 | 0.19367039 |
| 33 | 0.5 | 0.3 | 0.25 | 0.125 | 634 | 588744 | 0.4751 | 342 | 5816 | 0.19366885 |
| 34 | 0.6 | 0.3 | 0.25 | 0.125 | 1359 | 329816 | 0.2662 | 789 | 1296 | 0.19365675 |
| 35 | 0.7 | 0.3 | 0.25 | 0.125 | - | - | - | - | - | - |
| 36 | 0.8 | 0.3 | 0.25 | 0.125 | - | - | - | - | - | - |
| 37 | 0 | 0 | 0.33 | 0.167 | 252 | 767419 | 0.6193 | 125 | 22431 | 0.25216588 |
| 38 | 0.1 | 0 | 0.33 | 0.167 | 291 | 749889 | 0.6052 | 146 | 18564 | 0.25216530 |
| 39 | 0.2 | 0 | 0.33 | 0.167 | 347 | 718696 | 0.5800 | 177 | 14406 | 0.25216576 |
| 40 | 0.3 | 0 | 0.33 | 0.167 | 438 | 661238 | 0.5336 | 229 | 9987 | 0.25216389 |
| 41 | 0.4 | 0 | 0.33 | 0.167 | 617 | 550688 | 0.4444 | 336 | 5477 | 0.25216110 |
| 42 | 0.5 | 0 | 0.33 | 0.167 | 1198 | 325543 | 0.2627 | 693 | 1456 | 0.25214347 |
| 43 | 0.6 | 0 | 0.33 | 0.167 | - | - | - | - | - | - |
| 44 | 0.7 | 0 | 0.33 | 0.167 | - | - | - | - | - | - |
| 45 | 0.8 | 0 | 0.33 | 0.167 | - | - | - | - | - | - |
| 46 | 0 | 0.3 | 0.33 | 0.167 | 252 | 767419 | 0.6193 | 125 | 22431 | 0.17651612 |
| 47 | 0.1 | 0.3 | 0.33 | 0.167 | 291 | 749889 | 0.6052 | 146 | 18564 | 0.17651571 |
| 48 | 0.2 | 0.3 | 0.33 | 0.167 | 347 | 718696 | 0.5800 | 177 | 14406 | 0.17651603 |
| 49 | 0.3 | 0.3 | 0.33 | 0.167 | 438 | 661238 | 0.5336 | 229 | 9987 | 0.17651472 |
| 50 | 0.4 | 0.3 | 0.33 | 0.167 | 617 | 550688 | 0.4444 | 336 | 5477 | 0.17651277 |
| 51 | 0.5 | 0.3 | 0.33 | 0.167 | 1198 | 325543 | 0.2627 | 693 | 1456 | 0.17650043 |
| 52 | 0.6 | 0.3 | 0.33 | 0.167 | - | - | - | - | - | - |
| 53 | 0.7 | 0.3 | 0.33 | 0.167 | - | - | - | - | - | - |
| 54 | 0.8 | 0.3 | 0.33 | 0.167 | - | - | - | - | - | - |
| 55 | 0 | 0 | 0.5 | 0.25 | 355 | 675642 | 0.5452 | 183 | 12763 | 0.21421446 |
| 56 | 0.1 | 0 | 0.5 | 0.25 | 437 | 613263 | 0.4949 | 232 | 9002 | 0.21421285 |
| 57 | 0.2 | 0 | 0.5 | 0.25 | 586 | 511880 | 0.4131 | 321 | 5266 | 0.21420988 |
| 58 | 0.3 | 0 | 0.5 | 0.25 | 968 | 341708 | 0.2758 | 553 | 1930 | 0.21419497 |
| 59 | 0.4 | 0 | 0.5 | 0.25 | 5685 | 43400 | 0.0350 | 3701 | 26 | 0.21241196 |
| 60 | 0.5 | 0 | 0.5 | 0.25 | - | - | - | - | - | - |
| 61 | 0.6 | 0 | 0.5 | 0.25 | - | - | - | - | - | - |
| 62 | 0.7 | 0 | 0.5 | 0.25 | - | - | - | - | - | - |
| 63 | 0.8 | 0 | 0.5 | 0.25 | - | - | - | - | - | - |
| 64 | 0 | 0.3 | 0.5 | 0.25 | 355 | 675642 | 0.5452 | 183 | 12763 | 0.14995012 |
| 65 | 0.1 | 0.3 | 0.5 | 0.25 | 437 | 613263 | 0.4949 | 232 | 9002 | 0.14994900 |
| 66 | 0.2 | 0.3 | 0.5 | 0.25 | 586 | 511880 | 0.4131 | 321 | 5266 | 0.14994692 |
| 67 | 0.3 | 0.3 | 0.5 | 0.25 | 968 | 341708 | 0.2758 | 553 | 1930 | 0.14993648 |
| 68 | 0.4 | 0.3 | 0.5 | 0.25 | 5685 | 43400 | 0.0350 | 3701 | 26 | 0.14868838 |
| 69 | 0.5 | 0.3 | 0.5 | 0.25 | - | - | - | - | - | - |
| 70 | 0.6 | 0.3 | 0.5 | 0.25 | - | - | - | - | - | - |
| 71 | 0.7 | 0.3 | 0.5 | 0.25 | - | - | - | - | - | - |
| 72 | 0.8 | 0.3 | 0.5 | 0.25 | - | - | - | - | - | - |

OD=outbreak duration. CNC= cumulative number of cases. TAR= total attack rate.

PD= peak date. NPC= number of peak cases. *f*= case fatality rate.

-= has been controlled
