# Supplementary material for: Effectiveness of potential antiviral treatments in COVID-19 transmission control: a modelling study
Source: Infect Dis Poverty. 2021 Apr 19;10:53. doi: 10.1186/s40249-021-00835-2 (PMC8054260; doi:10.1186/s40249-021-00835-2)
Supplement: Supplementary file 8 — Additional file 8: Table S6. The reduction rate of total attack rate (TAR). [file 40249_2021_835_MOESM8_ESM.docx]

**Additional Table 6 The reduction rate of total attack rate (TAR)**

|  | age 1 | age 2 | age 3 | age 4 |
| --- | --- | --- | --- | --- |
| z=0.3 | 0.00% | 0.00% | 0.00% | 0.00% |
| v=0.1 | 11.92% | 4.52% | 1.10% | 0.30% |
| v=0.2 | 24.18% | 10.67% | 2.96% | 0.92% |
| v=0.3 | 36.94% | 19.11% | 6.23% | 2.25% |
| v=0.4 | 50.41% | 30.77% | 12.13% | 5.24% |
| v=0.5 | 64.84% | 46.81% | 23.25% | 12.38% |
| v=0.6 | 80.78% | 68.35% | 45.21% | 30.97% |
| v=0.7 | - | 95.78% | 90.65% | 85.94% |
| 1/y=4 | 19.20% | 8.24% | 2.11% | 0.62% |
| 1/y=3 | 41.00% | 23.17% | 7.83% | 2.94% |
| 1/y=2 | 67.46% | 51.74% | 26.70% | 14.55% |
| z=0.3 and v=0.1 | 11.92% | 4.52% | 1.10% | 0.30% |
| z=0.3 and v=0.2 | 24.18% | 10.67% | 2.96% | 0.92% |
| z=0.3 and v=0.3 | 36.94% | 19.11% | 6.23% | 2.25% |
| z=0.3 and v=0.4 | 50.41% | 30.77% | 12.13% | 5.24% |
| z=0.3 and v=0.5 | 64.84% | 46.81% | 23.25% | 12.38% |
| z=0.3 and v=0.6 | - | 68.35% | 45.21% | 30.97% |
| z=0.3 and v=0.7 | - | 95.78% | 90.65% | 85.94% |
| z=0.3 and 1/y=4 | 19.20% | 8.24% | 2.11% | 0.62% |
| z=0.3 and 1/y=3 | 41.00% | 23.17% | 7.83% | 2.94% |
| z=0.3 and 1/y=2 | 67.46% | 51.74% | 26.70% | 14.55% |
| 1/y=4 and v=0.1 | 29.70% | 14.35% | 4.20% | 1.38% |
| 1/y=4 and v=0.2 | 40.59% | 22.32% | 7.58% | 2.86% |
| 1/y=4 and v=0.3 | 52.02% | 32.75% | 13.17% | 5.80% |
| 1/y=4 and v=0.4 | 64.13% | 46.35% | 22.70% | 11.93% |
| 1/y=4 and v=0.5 | 77.28% | 63.73% | 39.50% | 25.54% |
| 1/y=4 and v=0.6 | 92.50% | 85.20% | 70.10% | 58.29% |
| 1/y=4 and v=0.7 | - | - | - | - |
| 1/y=3 and v=0.1 | 50.06% | 31.34% | 12.13% | 5.16% |
| 1/y=3 and v=0.2 | 59.49% | 41.39% | 18.67% | 9.10% |
| 1/y=3 and v=0.3 | 69.50% | 53.66% | 28.80% | 16.37% |
| 1/y=3 and v=0.4 | 80.22% | 68.37% | 44.81% | 30.35% |
| 1/y=3 and v=0.5 | 92.42% | 85.60% | 70.66% | 58.83% |
| 1/y=3 and v=0.6 | - | - | - | - |
| 1/y=3 and v=0.7 | - | - | - | - |
| 1/y=2 and v=0.1 | 74.89% | 61.55% | 36.46% | 22.44% |
| 1/y=2 and v=0.2 | 82.80% | 72.60% | 50.07% | 35.26% |
| 1/y=2 and v=0.3 | 91.47% | 84.89% | 69.23% | 56.78% |
| 1/y=2 and v=0.4 | - | 98.48% | 96.48% | 94.51% |
| 1/y=2 and v=0.5 | - | - | - | - |
| 1/y=2 and v=0.6 | - | - | - | - |
| 1/y=2 and v=0.7 | - | - | - | - |
| 1/y=4, z=0.3 and v=0.1 | 29.70% | 14.35% | 4.20% | 1.38% |
| 1/y=4, z=0.3 and v=0.2 | 40.59% | 22.32% | 7.58% | 2.86% |
| 1/y=4, z=0.3 and v=0.3 | 52.02% | 32.75% | 13.17% | 5.80% |
| 1/y=4, z=0.3 and v=0.4 | 64.13% | 46.35% | 22.70% | 11.93% |
| 1/y=4, z=0.3 and v=0.5 | 77.28% | 63.73% | 39.50% | 25.54% |
| 1/y=4, z=0.3 and v=0.6 | 92.50% | 85.20% | 70.10% | 58.29% |
| 1/y=4, z=0.3 and v=0.7 | - | - | - | - |
| 1/y=3, z=0.3 and v=0.1 | 50.06% | 31.34% | 12.13% | 5.16% |
| 1/y=3, z=0.3 and v=0.2 | 59.49% | 41.39% | 18.67% | 9.10% |
| 1/y=3, z=0.3 and v=0.3 | 69.50% | 53.66% | 28.80% | 16.37% |
| 1/y=3, z=0.3 and v=0.4 | 80.22% | 68.37% | 44.81% | 30.35% |
| 1/y=3, z=0.3 and v=0.5 | 92.42% | 85.60% | 70.66% | 58.83% |
| 1/y=3, z=0.3 and v=0.6 | - | - | - | - |
| 1/y=3, z=0.3 and v=0.7 | - | - | - | - |
| 1/y=2, z=0.3 and v=0.1 | 74.89% | 61.55% | 36.46% | 22.44% |
| 1/y=2, z=0.3 and v=0.2 | 82.80% | 72.60% | 50.07% | 35.26% |
| 1/y=2, z=0.3 and v=0.3 | 91.47% | 84.89% | 69.23% | 56.78% |
| 1/y=2, z=0.3 and v=0.4 | - | 98.48% | 96.48% | 94.51% |
| 1/y=2, z=0.3 and v=0.5 | - | - | - | - |
| 1/y=2, z=0.3 and v=0.6 | - | - | - | - |
| 1/y=2, z=0.3 and v=0.7 | - | - | - | - |

1/age 1: ≤ 14 years; age 2: 15–44 years; age 3: 45–64 years; age 4: ≥ 65 years1/.
