# Supplementary material for: Effectiveness of potential antiviral treatments in COVID-19 transmission control: a modelling study
Source: Infect Dis Poverty. 2021 Apr 19;10:53. doi: 10.1186/s40249-021-00835-2 (PMC8054260; doi:10.1186/s40249-021-00835-2)
Supplement: Supplementary file 9 — Additional file 9: Table S7. The absolute reduction value of total attack rate (TAR). [file 40249_2021_835_MOESM9_ESM.docx]

**Additional Table 7** **The absolute reduction** **value of total attack rate (TAR)**

|  | age 1 | age 2 | age 3 | age 4 |
| --- | --- | --- | --- | --- |
| z=0.3 | 0.0000 | 0.0000 | 0.0000 | 0.0000 |
| v=0.1 | 0.0004 | 0.0253 | 0.0069 | 0.0019 |
| v=0.2 | 0.0009 | 0.0598 | 0.0186 | 0.0059 |
| v=0.3 | 0.0014 | 0.1070 | 0.0392 | 0.0144 |
| v=0.4 | 0.0019 | 0.1723 | 0.0763 | 0.0335 |
| v=0.5 | 0.0024 | 0.2622 | 0.1463 | 0.0790 |
| v=0.6 | 0.0030 | 0.3828 | 0.2845 | 0.1976 |
| v=0.7 | - | 0.5365 | 0.5704 | 0.5484 |
| 1/y=4 | 0.0007 | 0.0462 | 0.0133 | 0.0039 |
| 1/y=3 | 0.0015 | 0.1298 | 0.0493 | 0.0188 |
| 1/y=2 | 0.0025 | 0.2898 | 0.1680 | 0.0928 |
| z=0.3 and v=0.1 | 0.0004 | 0.0253 | 0.0069 | 0.0019 |
| z=0.3 and v=0.2 | 0.0009 | 0.0598 | 0.0186 | 0.0059 |
| z=0.3 and v=0.3 | 0.0014 | 0.1070 | 0.0392 | 0.0144 |
| z=0.3 and v=0.4 | 0.0019 | 0.1723 | 0.0763 | 0.0335 |
| z=0.3 and v=0.5 | 0.0024 | 0.2622 | 0.1463 | 0.0790 |
| z=0.3 and v=0.6 | - | 0.3828 | 0.2845 | 0.1976 |
| z=0.3 and v=0.7 | - | 0.5365 | 0.5704 | 0.5484 |
| z=0.3 and 1/y=4 | 0.0007 | 0.0462 | 0.0133 | 0.0039 |
| z=0.3 and 1/y=3 | 0.0015 | 0.1298 | 0.0493 | 0.0188 |
| z=0.3 and 1/y=2 | 0.0025 | 0.2898 | 0.1680 | 0.0928 |
| 1/y=4 and v=0.1 | 0.0011 | 0.0804 | 0.0264 | 0.0088 |
| 1/y=4 and v=0.2 | 0.0015 | 0.1250 | 0.0477 | 0.0182 |
| 1/y=4 and v=0.3 | 0.0019 | 0.1834 | 0.0828 | 0.0370 |
| 1/y=4 and v=0.4 | 0.0024 | 0.2596 | 0.1428 | 0.0761 |
| 1/y=4 and v=0.5 | 0.0029 | 0.3570 | 0.2485 | 0.1629 |
| 1/y=4 and v=0.6 | 0.0034 | 0.4772 | 0.4411 | 0.3719 |
| 1/y=4 and v=0.7 | - | - | - | - |
| 1/y=3 and v=0.1 | 0.0019 | 0.1755 | 0.0763 | 0.0329 |
| 1/y=3 and v=0.2 | 0.0022 | 0.2318 | 0.1175 | 0.0581 |
| 1/y=3 and v=0.3 | 0.0026 | 0.3005 | 0.1812 | 0.1044 |
| 1/y=3 and v=0.4 | 0.0030 | 0.3829 | 0.2820 | 0.1937 |
| 1/y=3 and v=0.5 | 0.0034 | 0.4794 | 0.4446 | 0.3753 |
| 1/y=3 and v=0.6 | - | - | - | - |
| 1/y=3 and v=0.7 | - | - | - | - |
| 1/y=2 and v=0.1 | 0.0028 | 0.3448 | 0.2294 | 0.1432 |
| 1/y=2 and v=0.2 | 0.0031 | 0.4066 | 0.3151 | 0.2250 |
| 1/y=2 and v=0.3 | 0.0034 | 0.4755 | 0.4356 | 0.3623 |
| 1/y=2 and v=0.4 | - | 0.5515 | 0.6070 | 0.6030 |
| 1/y=2 and v=0.5 | - | - | - | - |
| 1/y=2 and v=0.6 | - | - | - | - |
| 1/y=2 and v=0.7 | - | - | - | - |
| 1/y=4, z=0.3 and v=0.1 | 0.0011 | 0.0804 | 0.0264 | 0.0088 |
| 1/y=4, z=0.3 and v=0.2 | 0.0015 | 0.1250 | 0.0477 | 0.0182 |
| 1/y=4, z=0.3 and v=0.3 | 0.0019 | 0.1834 | 0.0828 | 0.0370 |
| 1/y=4, z=0.3 and v=0.4 | 0.0024 | 0.2596 | 0.1428 | 0.0761 |
| 1/y=4, z=0.3 and v=0.5 | 0.0029 | 0.3570 | 0.2485 | 0.1629 |
| 1/y=4, z=0.3 and v=0.6 | 0.0034 | 0.4772 | 0.4411 | 0.3719 |
| 1/y=4, z=0.3 and v=0.7 | - | - | - | - |
| 1/y=3, z=0.3 and v=0.1 | 0.0019 | 0.1755 | 0.0763 | 0.0329 |
| 1/y=3, z=0.3 and v=0.2 | 0.0022 | 0.2318 | 0.1175 | 0.0581 |
| 1/y=3, z=0.3 and v=0.3 | 0.0026 | 0.3005 | 0.1812 | 0.1044 |
| 1/y=3, z=0.3 and v=0.4 | 0.0030 | 0.3829 | 0.2820 | 0.1937 |
| 1/y=3, z=0.3 and v=0.5 | 0.0034 | 0.4794 | 0.4446 | 0.3753 |
| 1/y=3, z=0.3 and v=0.6 | - | - | - | - |
| 1/y=3, z=0.3 and v=0.7 | - | - | - | - |
| 1/y=2, z=0.3 and v=0.1 | 0.0028 | 0.3448 | 0.2294 | 0.1432 |
| 1/y=2, z=0.3 and v=0.2 | 0.0031 | 0.4066 | 0.3151 | 0.2250 |
| 1/y=2, z=0.3 and v=0.3 | 0.0034 | 0.4755 | 0.4356 | 0.3623 |
| 1/y=2, z=0.3 and v=0.4 | - | 0.5515 | 0.6070 | 0.6030 |
| 1/y=2, z=0.3 and v=0.5 | - | - | - | - |
| 1/y=2, z=0.3 and v=0.6 | - | - | - | - |
| 1/y=2, z=0.3 and v=0.7 | - | - | - | - |

age 1: ≤ 14 years; age 2: 15–44 years; age 3: 45–64 years; age 4: ≥ 65 years.

**Additional Table 9 The reduction absolute value of case fatality rate (*f*)**

|  | age 1 | age 2 | age 3 | age 4 |
| --- | --- | --- | --- | --- |
| z=0.3 | 0.0021 | 0.0059 | 0.0296 | 0.0881 |
| v=0.1 | 0.0000 | 0.0000 | 0.0000 | 0.0000 |
| v=0.2 | 0.0001 | 0.0000 | 0.0000 | 0.0000 |
| v=0.3 | 0.0001 | 0.0000 | 0.0000 | 0.0000 |
| v=0.4 | 0.0003 | 0.0000 | 0.0000 | 0.0000 |
| v=0.5 | 0.0006 | 0.0000 | 0.0000 | 0.0000 |
| v=0.6 | - | 0.0000 | 0.0000 | 0.0000 |
| v=0.7 | - | 0.0001 | 0.0001 | 0.0002 |
| 1/y=4 | 0.0013 | 0.0026 | 0.0101 | 0.0171 |
| 1/y=3 | 0.0027 | 0.0057 | 0.0231 | 0.0416 |
| 1/y=2 | 0.0046 | 0.0095 | 0.0402 | 0.0796 |
| z=0.3 and v=0.1 | 0.0021 | 0.0059 | 0.0296 | 0.0881 |
| z=0.3 and v=0.2 | 0.0022 | 0.0059 | 0.0296 | 0.0881 |
| z=0.3 and v=0.3 | 0.0022 | 0.0059 | 0.0296 | 0.0881 |
| z=0.3 and v=0.4 | 0.0023 | 0.0059 | 0.0296 | 0.0881 |
| z=0.3 and v=0.5 | 0.0025 | 0.0059 | 0.0296 | 0.0881 |
| z=0.3 and v=0.6 | - | 0.0059 | 0.0296 | 0.0881 |
| z=0.3 and v=0.7 | - | 0.0060 | 0.0297 | 0.0883 |
| z=0.3 and 1/y=4 | 0.0030 | 0.0077 | 0.0367 | 0.1001 |
| z=0.3 and 1/y=3 | 0.0040 | 0.0099 | 0.0457 | 0.1173 |
| z=0.3 and 1/y=2 | 0.0053 | 0.0125 | 0.0577 | 0.1439 |
| 1/y=4 and v=0.1 | 0.0013 | 0.0026 | 0.0101 | 0.0171 |
| 1/y=4 and v=0.2 | 0.0014 | 0.0026 | 0.0101 | 0.0171 |
| 1/y=4 and v=0.3 | 0.0015 | 0.0026 | 0.0101 | 0.0171 |
| 1/y=4 and v=0.4 | 0.0018 | 0.0026 | 0.0101 | 0.0171 |
| 1/y=4 and v=0.5 | 0.0029 | 0.0026 | 0.0101 | 0.0171 |
| 1/y=4 and v=0.6 | - | 0.0026 | 0.0101 | 0.0172 |
| 1/y=4 and v=0.7 | - | - | - | - |
| 1/y=3 and v=0.1 | 0.0028 | 0.0057 | 0.0231 | 0.0416 |
| 1/y=3 and v=0.2 | 0.0030 | 0.0057 | 0.0231 | 0.0416 |
| 1/y=3 and v=0.3 | 0.0033 | 0.0057 | 0.0231 | 0.0416 |
| 1/y=3 and v=0.4 | - | 0.0057 | 0.0231 | 0.0416 |
| 1/y=3 and v=0.5 | - | 0.0058 | 0.0231 | 0.0417 |
| 1/y=3 and v=0.6 | - | - | - | - |
| 1/y=3 and v=0.7 | - | - | - | - |
| 1/y=2 and v=0.1 | 0.0054 | 0.0095 | 0.0402 | 0.0796 |
| 1/y=2 and v=0.2 | - | 0.0095 | 0.0402 | 0.0796 |
| 1/y=2 and v=0.3 | - | 0.0095 | 0.0402 | 0.0796 |
| 1/y=2 and v=0.4 | - | 0.0099 | 0.0409 | 0.0814 |
| 1/y=2 and v=0.5 | - | - | - | - |
| 1/y=2 and v=0.6 | - | - | - | - |
| 1/y=2 and v=0.7 | - | - | - | - |
| 1/y=4, z=0.3 and v=0.1 | 0.0031 | 0.0077 | 0.0367 | 0.1001 |
| 1/y=4, z=0.3 and v=0.2 | 0.0031 | 0.0077 | 0.0367 | 0.1001 |
| 1/y=4, z=0.3 and v=0.3 | 0.0032 | 0.0077 | 0.0367 | 0.1001 |
| 1/y=4, z=0.3 and v=0.4 | 0.0034 | 0.0077 | 0.0367 | 0.1001 |
| 1/y=4, z=0.3 and v=0.5 | 0.0042 | 0.0077 | 0.0367 | 0.1001 |
| 1/y=4, z=0.3 and v=0.6 | - | 0.0078 | 0.0367 | 0.1001 |
| 1/y=4, z=0.3 and v=0.7 | - | - | - | - |
| 1/y=3, z=0.3 and v=0.1 | 0.0041 | 0.0099 | 0.0457 | 0.1173 |
| 1/y=3, z=0.3 and v=0.2 | 0.0042 | 0.0099 | 0.0457 | 0.1173 |
| 1/y=3, z=0.3 and v=0.3 | 0.0044 | 0.0099 | 0.0457 | 0.1173 |
| 1/y=3, z=0.3 and v=0.4 | - | 0.0099 | 0.0457 | 0.1173 |
| 1/y=3, z=0.3 and v=0.5 | - | 0.0099 | 0.0457 | 0.1173 |
| 1/y=3, z=0.3 and v=0.6 | - | - | - | - |
| 1/y=3, z=0.3 and v=0.7 | - | - | - | - |
| 1/y=2, z=0.3 and v=0.1 | 0.0059 | 0.0125 | 0.0577 | 0.1439 |
| 1/y=2, z=0.3 and v=0.2 | - | 0.0125 | 0.0577 | 0.1439 |
| 1/y=2, z=0.3 and v=0.3 | - | 0.0125 | 0.0577 | 0.1439 |
| 1/y=2, z=0.3 and v=0.4 | - | 0.0128 | 0.0582 | 0.1451 |
| 1/y=2, z=0.3 and v=0.5 | - | - | - | - |
| 1/y=2, z=0.3 and v=0.6 | - | - | - | - |
| 1/y=2, z=0.3 and v=0.7 | - | - | - | - |

age 1: ≤ 14 years; age 2: 15–44 years; age 3: 45–64 years; age 4: ≥ 65 years.
