# Supplementary material for: Effectiveness of potential antiviral treatments in COVID-19 transmission control: a modelling study
Source: Infect Dis Poverty. 2021 Apr 19;10:53. doi: 10.1186/s40249-021-00835-2 (PMC8054260; doi:10.1186/s40249-021-00835-2)
Supplement: Supplementary file 10 — Additional file 10: Table S8. The reduction rate of case fatality rate (f). [file 40249_2021_835_MOESM10_ESM.docx]

**Additional Table 8 The reduction rate of case fatality rate (*f*)**

|  | age 1 | age 2 | age 3 | age 4 |
| --- | --- | --- | --- | --- |
| z=0.3 | 30.0000% | 30.0000% | 30.0000% | 30.0000% |
| v=0.1 | 0.4215% | -0.0001% | 0.0002% | 0.0000% |
| v=0.2 | 0.9264% | 0.0001% | 0.0002% | 0.0002% |
| v=0.3 | 2.0587% | 0.0005% | 0.0002% | 0.0003% |
| v=0.4 | 3.9199% | 0.0008% | 0.0006% | 0.0004% |
| v=0.5 | 8.5013% | 0.0019% | 0.0010% | 0.0005% |
| v=0.6 | - | 0.0062% | 0.0026% | 0.0020% |
| v=0.7 | - | 0.3404% | 0.1092% | 0.0746% |
| 1/y=4 | 18.3045% | 13.3903% | 10.2729% | 5.8313% |
| 1/y=3 | 38.5295% | 29.1928% | 23.3900% | 14.1727% |
| 1/y=2 | 64.5572% | 48.1246% | 40.7220% | 27.0899% |
| z=0.3 and v=0.1 | 30.2950% | 29.9999% | 30.0001% | 30.0000% |
| z=0.3 and v=0.2 | 30.6485% | 30.0001% | 30.0002% | 30.0001% |
| z=0.3 and v=0.3 | 31.4411% | 30.0003% | 30.0002% | 30.0002% |
| z=0.3 and v=0.4 | 32.7440% | 30.0006% | 30.0004% | 30.0002% |
| z=0.3 and v=0.5 | 35.9509% | 30.0013% | 30.0007% | 30.0004% |
| z=0.3 and v=0.6 | - | 30.0043% | 30.0019% | 30.0014% |
| z=0.3 and v=0.7 | - | 30.2383% | 30.0765% | 30.0523% |
| z=0.3 and 1/y=4 | 42.8132% | 39.3732% | 37.1911% | 34.0819% |
| z=0.3 and 1/y=3 | 56.9706% | 50.4350% | 46.3730% | 39.9209% |
| z=0.3 and 1/y=2 | 75.1900% | 63.6872% | 58.5054% | 48.9629% |
| 1/y=4 and v=0.1 | 19.0508% | 13.3905% | 10.2731% | 5.8313% |
| 1/y=4 and v=0.2 | 19.7943% | 13.3906% | 10.2731% | 5.8313% |
| 1/y=4 and v=0.3 | 21.5675% | 13.3911% | 10.2734% | 5.8316% |
| 1/y=4 and v=0.4 | 25.1168% | 13.3919% | 10.2738% | 5.8318% |
| 1/y=4 and v=0.5 | 41.2342% | 13.3944% | 10.2748% | 5.8326% |
| 1/y=4 and v=0.6 | - | 13.4165% | 10.2824% | 5.8385% |
| 1/y=4 and v=0.7 | - | - | - | - |
| 1/y=3 and v=0.1 | 39.5275% | 29.1932% | 23.3900% | 14.1729% |
| 1/y=3 and v=0.2 | 41.9346% | 29.1936% | 23.3903% | 14.1728% |
| 1/y=3 and v=0.3 | 46.7618% | 29.1947% | 23.3907% | 14.1734% |
| 1/y=3 and v=0.4 | - | 29.1978% | 23.3919% | 14.1744% |
| 1/y=3 and v=0.5 | - | 29.2187% | 23.3993% | 14.1804% |
| 1/y=3 and v=0.6 | - | - | - | - |
| 1/y=3 and v=0.7 | - | - | - | - |
| 1/y=2 and v=0.1 | 75.8246% | 48.1260% | 40.7226% | 27.0904% |
| 1/y=2 and v=0.2 | - | 48.1293% | 40.7240% | 27.0915% |
| 1/y=2 and v=0.3 | - | 48.1442% | 40.7296% | 27.0965% |
| 1/y=2 and v=0.4 | - | 50.2874% | 41.4586% | 27.7034% |
| 1/y=2 and v=0.5 | - | - | - | - |
| 1/y=2 and v=0.6 | - | - | - | - |
| 1/y=2 and v=0.7 | - | - | - | - |
| 1/y=4, z=0.3 and v=0.1 | 43.3355% | 39.3733% | 37.1912% | 34.0819% |
| 1/y=4, z=0.3 and v=0.2 | 43.8560% | 39.3735% | 37.1912% | 34.0819% |
| 1/y=4, z=0.3 and v=0.3 | 45.0972% | 39.3737% | 37.1914% | 34.0821% |
| 1/y=4, z=0.3 and v=0.4 | 47.5818% | 39.3743% | 37.1916% | 34.0823% |
| 1/y=4, z=0.3 and v=0.5 | 58.8639% | 39.3761% | 37.1924% | 34.0828% |
| 1/y=4, z=0.3 and v=0.6 | - | 39.3915% | 37.1977% | 34.0869% |
| 1/y=4, z=0.3 and v=0.7 | - | - | - | - |
| 1/y=3, z=0.3 and v=0.1 | 57.6693% | 50.4352% | 46.3730% | 39.9211% |
| 1/y=3, z=0.3 and v=0.2 | 59.3542% | 50.4355% | 46.3732% | 39.9209% |
| 1/y=3, z=0.3 and v=0.3 | 62.7333% | 50.4363% | 46.3735% | 39.9214% |
| 1/y=3, z=0.3 and v=0.4 | - | 50.4385% | 46.3744% | 39.9221% |
| 1/y=3, z=0.3 and v=0.5 | - | 50.4531% | 46.3795% | 39.9263% |
| 1/y=3, z=0.3 and v=0.6 | - | - | - | - |
| 1/y=3, z=0.3 and v=0.7 | - | - | - | - |
| 1/y=2, z=0.3 and v=0.1 | 83.0772% | 63.6882% | 58.5059% | 48.9633% |
| 1/y=2, z=0.3 and v=0.2 | - | 63.6905% | 58.5068% | 48.9640% |
| 1/y=2, z=0.3 and v=0.3 | - | 63.7009% | 58.5107% | 48.9676% |
| 1/y=2, z=0.3 and v=0.4 | - | 65.2012% | 59.0210% | 49.3924% |
| 1/y=2, z=0.3 and v=0.5 | - | - | - | - |
| 1/y=2, z=0.3 and v=0.6 | - | - | - | - |
| 1/y=2, z=0.3 and v=0.7 | - | - | - | - |

age 1: ≤ 14 years; age 2: 15–44 years; age 3: 45–64 years; age 4: ≥ 65 years.
